# Establishment of an Arabidopsis callus system to study the interrelations of biosynthesis, degradation and accumulation of carotenoids

**DOI:** 10.1371/journal.pone.0192158

**Published:** 2018-02-02

**Authors:** Patrick Schaub, Marta Rodriguez-Franco, Christopher Ian Cazzonelli, Daniel Álvarez, Florian Wüst, Ralf Welsch

**Affiliations:** 1 University of Freiburg, Faculty of Biology, Institute for Biology II, Freiburg, Germany; 2 Hawkesbury Institute for the Environment, University of Western Sydney, Hawkesbury Campus, Richmond, NSW Australia; Zhejiang University, CHINA

## Abstract

The net amounts of carotenoids accumulating in plant tissues are determined by the rates of biosynthesis and degradation. While biosynthesis is rate-limited by the activity of PHYTOENE SYNTHASE (PSY), carotenoid losses are caused by catabolic enzymatic and non-enzymatic degradation. We established a system based on non-green Arabidopsis callus which allowed investigating major determinants for high steady-state levels of β-carotene. Wild-type callus development was characterized by strong carotenoid degradation which was only marginally caused by the activity of carotenoid cleavage oxygenases. In contrast, carotenoid degradation occurred mostly non-enzymatically and selectively affected carotenoids in a molecule-dependent manner. Using carotenogenic pathway mutants, we found that linear carotenes such as phytoene, phytofluene and pro-lycopene resisted degradation and accumulated while β-carotene was highly susceptible towards degradation. Moderately increased pathway activity through *PSY* overexpression was compensated by degradation revealing no net increase in β-carotene. However, higher pathway activities outcompeted carotenoid degradation and efficiently increased steady-state β-carotene amounts to up to 500 μg g^-1^ dry mass. Furthermore, we identified oxidative β-carotene degradation products which correlated with pathway activities, yielding β-apocarotenals of different chain length and various apocarotene-dialdehydes. The latter included methylglyoxal and glyoxal as putative oxidative end products suggesting a potential recovery of carotenoid-derived carbon for primary metabolic pathways. Moreover, we investigated the site of β-carotene sequestration by co-localization experiments which revealed that β-carotene accumulated as intra-plastid crystals which was confirmed by electron microscopy with carotenoid-accumulating roots. The results are discussed in the context of using the non-green calli carotenoid assay system for approaches targeting high steady-state β-carotene levels prior to their application in crops.

## Introduction

Carotenoids are plastid-synthesized lipophilic isoprenoids which serve as precursors for pharmacologically relevant metabolites, such as crocetin or β-cryptoxanthin [1–3]. Moreover, animals including humans rely on carotenoid uptake through their diet to exploit their function as radical scavenger and provitamin A [4]. Accordingly, there are numerous approaches to increase the carotenoid content of various crops to fight vitamin A deficiencies [5]. Most provitamin A-rich edible tissues, such as fruits and seeds, are non-green, which restricts the use of Arabidopsis as a model system for basic research. In this study we established an Arabidopsis-derived, non-green callus assay to develop strategies for increasing and stabilizing β-carotene which can also be applied for carotenoid-derived phytochemicals. Serving as a model, the cell culture system allows evaluating suitable gene combinations to be tested prior to engineering biofortified crops.

Plant tissue cultures are frequently used as a scalable alternative to whole plants as a source for plant secondary metabolites for pharmaceuticals, flavours, colours and food additives. Preferably, cell suspension cultures are exploited to produce phytochemicals, including phenylpropanoids, alkaloids, steroids, quinines and terpenoids [6–8]. Product specificity and yield is often limited by weak expression of relevant pathway genes in undifferentiated callus cells and by intracellular degradation of desired compounds. While biotechnological approaches are frequently used to specifically adopt pathway fluxes, stability issues can only be overcome by *in situ* product removal requiring solubility and secretion [8,9]. While this applies for some compounds like resveratrol, it is unsuitable for lipophilic metabolites that are synthesized and stored within plant cells.

In non-photosynthetic tissues, coloured carotenoids are sequestered in diverse sub-organellar structures of chromoplasts. Depending on the sequestration mode, chromoplasts are classified as globular, crystalline, membranous, fibrillar and tubular [10,11]. Tissue type, developmental and environmental conditions determine carotenoid losses by enzymatic and non-enzymatic processes. Thus, carotenoid accumulation is determined by the relative rates of synthesis and degradation [12,13]. In leaf chloroplasts carotenoids are indispensable constituents of photosynthesis [14,15]. In some plastid types carotenoids are precursors for signalling molecules and hormones that affect gene regulatory, physiological and developmental processes [16].

**Fig 1:**
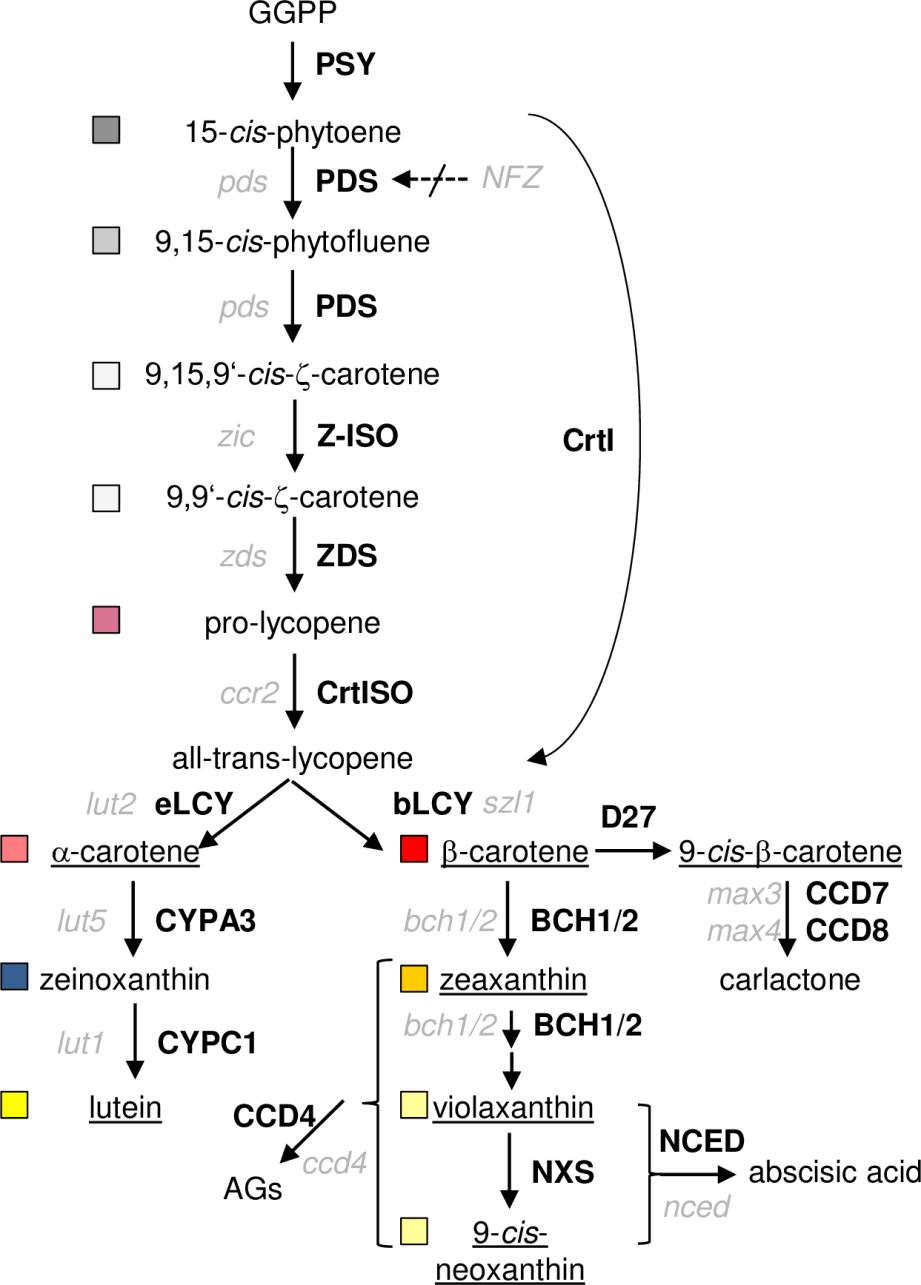
Carotenoid synthesis and cleavage pathway in higher plants. Enzymes (in bold) and corresponding Arabidopsis mutant names (in italics) are given; carotenoids accumulating in WT tissues are underlined; boxes indicate colour code used for carotenoids in subsequent figures. GGPP, geranylgeranyl diphosphate; PSY, phytoene synthase; PDS, phytoene desaturase; Z-ISO, ζ-carotene isomerase; ZDS, ζ-carotene desaturase; CRTISO, carotenoid isomerase; eLCY, ε-cyclase; bLCY, β-cyclase; CYPA3, cytP450 hydroxylase 97A3; CYPC1, cytP450 hydroxylase 97C1; BCH1/2, β-carotene hydroxylase 1/2; NXS, neoxanthin synthase; NCED, 9-*cis*-epoxycarotenoid dioxygenase; CCD, carotenoid cleavage dioxygenase; D27, β-carotene isomerase; CrtI, bacterial carotene desaturase; AGs, apocarotenoid glucosides; NFZ, norflurazon.

The rate of carotenoid biosynthesis is mainly determined by the first committed enzyme of the pathway, phytoene synthase (PSY; Fig 1) [17,18]. Phytoene is desaturated to ζ-carotene and pro-lycopene by phytoene desaturase (PDS) and ζ-carotene desaturase (ZDS), respectively. Two *cis-trans* carotenoid isomerases are required to isomerize the central 15-*cis* double bond in phytoene and the *cis*-configured double bonds in pro-lycopene to form all-*trans*-lycopene. These reactions are catalyzed by Z-ISO and CRTISO, respectively, but substitutable by photoisomerization in association with a functional photosynthetic apparatus [19–23]. Therefore, *cis*-configured intermediates accumulate predominantly in etiolated tissues of *crtISO* mutants (*tangerine*, tomato; *ccr2*, Arabidopsis) and *Z-ISO* mutants (*y9*, maize; *zic*, Arabidopsis).

Two cyclases introduce terminal ε- or β-ionone rings into all-*trans*-lycopene, resulting in α-(ε,β-) or β-(β,β)-carotene, respectively. Hydroxylations yields the xanthophylls lutein from α-carotene and zeaxanthin from β-carotene [24] catalyzed by five carotene hydroxylases in Arabidopsis including the three cytochrome p450 enzymes CYP97A3/LUT5, CYP97C1/LUT1 and CYP97B3 which was identified only recently [25,26]. Zeaxanthin mono- and diepoxidation catalyzed by zeaxanthin epoxidase yields antheraxanthin and violaxanthin, respectively. The reverse reaction catalyzed by violaxanthin deepoxidase represents an inducible photoprotective mechanism, the xanthophyll cycle. Finally, neoxanthin synthase catalyzes the conversion of violaxanthin into an allenic carotenoid.

The carotenoid backbone can be cleaved by enzymatic and non-enzymatic processes. The former is catalyzed by carotenoid cleavage dioxygenase/nine-*cis*-epoxy-carotenoid dioxygenases (CCD/NCED) [16,27]. In Arabidopsis, this gene family comprises nine members, five of which are assumed to be involved in abscisic acid synthesis (ABA; AtNCED2,3,5,6 and 9) [28,29]. Two CCDs are committed to strigolactone biosynthesis (CCD7/MAX3 and CCD8/MAX4) [30]. The two remaining CCD1 and CCD4 contribute to carotenoid degradation and cleave their substrates into volatile apocarotenoids, producing scents [31–33]. Arabidopsis seed carotenoid content is mainly determined by CCD4 with only minor contribution of CCD1, while CCD4 determines petal colour in *Chrysanthemum* and norisoprenoid formation in peaches [34–36].

Non-enzymatic carotenoid destruction is related to their antioxidant properties. Their highly unsaturated hydrocarbon backbone is oxidized to form epoxy- and peroxyl derivatives, decompose into apocarotenoids and finally form a plethora of products which are partially identical to those formed enzymatically [37,38]. In chloroplasts, photooxidation seemingly predominates and consumes carotenoids [12,13,39]. A variety of signalling apocarotenoids protect against high-light stress by regulating gene expression [40–42].

Although the instability of carotenoids is well known, data correlating carotenoid turnover rates with degradation products in living cells are sparse. Aiming at unravelling these mechanisms, we used Arabidopsis lines with enhanced carotenoid pathway activity obtained by overexpressing *PSY* which provoked pathway flux imbalances. We determined a concurrent high carotenoid degradation activity present in wild-type (WT) calli selectively affecting carotenoid molecules. Our results reveal that a constantly high pathway activity is required to counteract carotenoid degradation and to maintain high steady-state β-carotene levels. Furthermore, we identified β-carotene-derived turnover metabolites revealing a possible recovery route for carotenoid-derived carbon to feed back into primary metabolic pathways.

## Results

### Carotenoid degradation during Arabidopsis callus development

**Fig 2:**
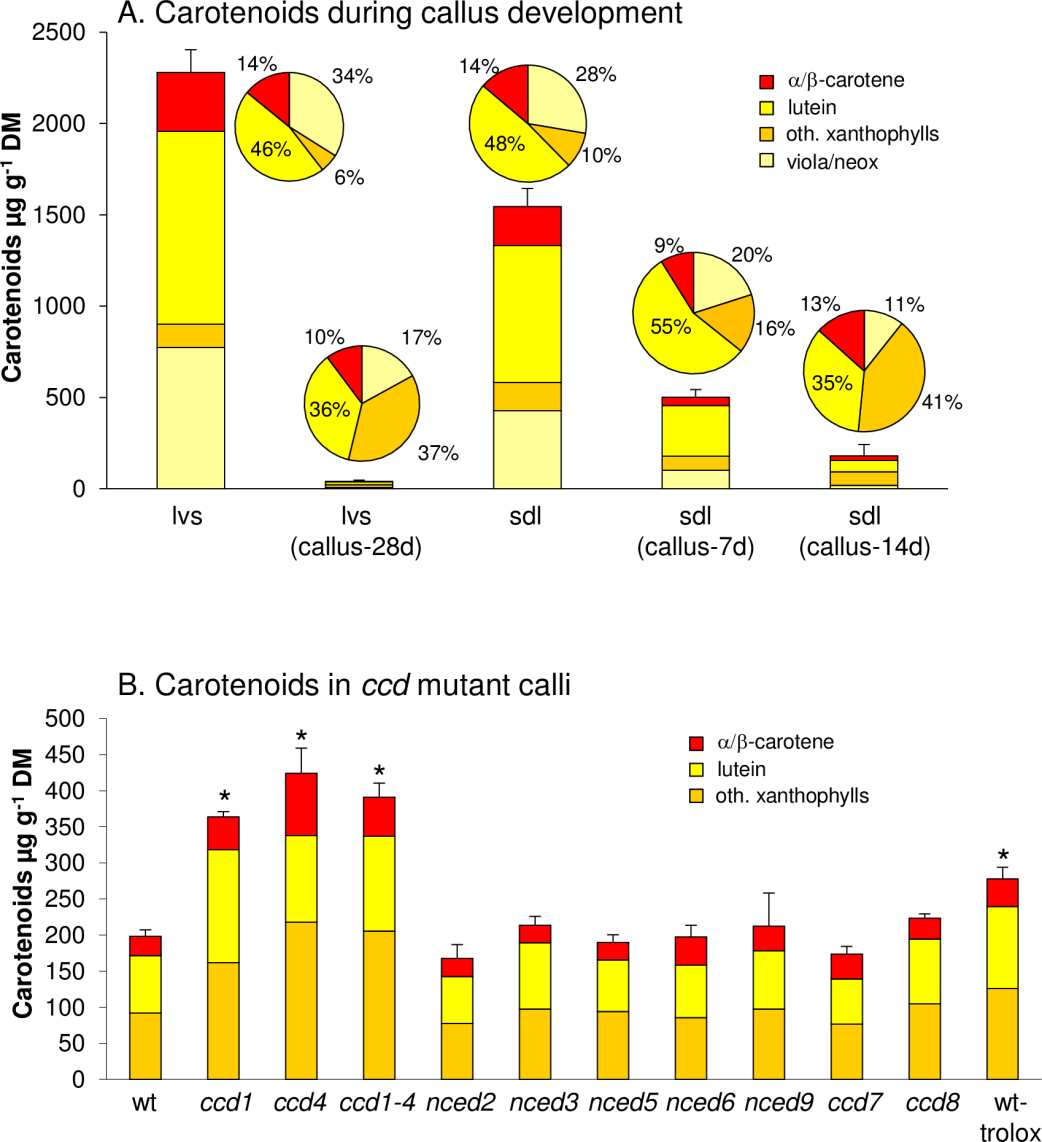
Carotenoid accumulation during Arabidopsis callus development. A, Carotenoid content in Arabidopsis leaves (lvs) decreases strongly in callus developed for 28 d on callus-inducing medium in darkness (CIM; lvs callus-28d). Seedlings light-germinated for 5 days on CIM (sdl) contain carotenoid levels almost similar to leaves and progressively lose carotenoids during 7 and 14 days of callus development in darkness (callus-7d/14d). Pie charts show carotenoid patterns. B, Carotenoid breakdown in Arabidopsis *CCD/NCED* mutants is reduced only in *ccd1*, *ccd4* and *ccd1 ccd4* double mutant and in WT callus generated in presence of the water-soluble tocopherol analogue trolox. Results are mean ± SD from at least three biological replicates. Significant difference to the wt, *P<0.05.

Callus cells form upon exposure of plant tissues on auxin/kinetin containing medium (callus inducing medium, CIM) and are considered pluripotent resembling meristematic root cells [43]. Carotenoid content in callus induced from Arabidopsis leaves incubated on CIM decreases dramatically to only trace amounts within four weeks incubation in darkness. Specifically, Arabidopsis WT leaves with about 2300 μg g^-1^ carotenoids (expressed per dry mass, DM) retain about 1% after four weeks of callus generation with only 40.8 μg g^-1^ residual carotenoid content remaining (Fig 2A; for detailed carotenoid data, see S1 Table).

In order to identify factors determining carotenoid breakdown, we applied a protocol which allowed developing calli directly from Arabidopsis seedlings. This protocol eliminates contamination-prone tissue subculturing and allows producing several grams of callus for analyses within 3 weeks. Seeds were germinated on CIM for 5 days under long-day conditions and thereafter incubated for 7 and 14 days in darkness [44]. During the initial illumination, seedlings developed green cotyledons and accumulated carotenoid and chlorophyll amounts only slightly lower than those in rosette leaves (Fig 2A). Calli developed for two weeks showed a considerable reduction in carotenoid levels with only about 11% remaining compared to initial carotenoid amounts, and with comparable carotenoid patterns like leaf-derived calli. Carotenoid losses after callus etiolation uniformly affected all major carotenoids present after 5 days illumination (violaxanthin and neoxanthin: 94.4%; lutein 90.2%, α-/β-carotene, 88.4%). Moreover, with the exception of xanthophyll esters consisting mainly of esterified antheraxanthin, we did not detect additional carotenoid species that were not present in leaves (see S1 Fig).

The contribution of enzyme-mediated carotenoid turnover was determined with Arabidopsis lines deficient in carotenoid cleavage dioxygenases. We generated calli from all Arabidopsis *CCD* null mutants, as well as from a *ccd1 ccd4* double mutant line [36]. Carotenoid levels were similar to WT calli in all lines albeit about two-fold increases in the *ccd1*, *ccd4* and *ccd1 ccd4* mutants were observed. Compared with carotenoids prior to etiolation, this corresponds to residual 20% while carotenoids in WT calli are reduced to only 11% (Fig 2B; see S2 Fig for mutant calli images). As carotenoid losses during callus development occurred largely without involvement of CCDs, we considered non-enzymatic carotenoid breakdown which is often counteracted by high abundance of antioxidants *in vitro* [45,46] and *in vivo* [47,48]. In fact, callus etiolation in presence of the water-soluble vitamin E analog trolox resulted in about 300 μg g^-1^ carotenoids, thus about 50% higher than in untreated controls, indicating a retarded carotenoid degradation (Fig 2B).

### Accumulation of pathway intermediates in carotenoid biosynthesis mutants

We questioned whether oxidation equally affects carotenoid species which normally do not accumulate in WT calli and analyzed calli from Arabidopsis mutants exhibiting different leaf carotenoid patterns. We focussed on characterized cytochrome p450 carotene hydroxylase mutants: *lut1* which is devoid of lutein but still accumulates the mono-hydroxylated α-carotene precursor zeinoxanthin and *lut5* which accumulates α-carotene [24,49,50]. Moreover, we included the *crtISO* mutant, which accumulates *cis*-carotene intermediates in the absence of light [20,51].

**Fig 3:**
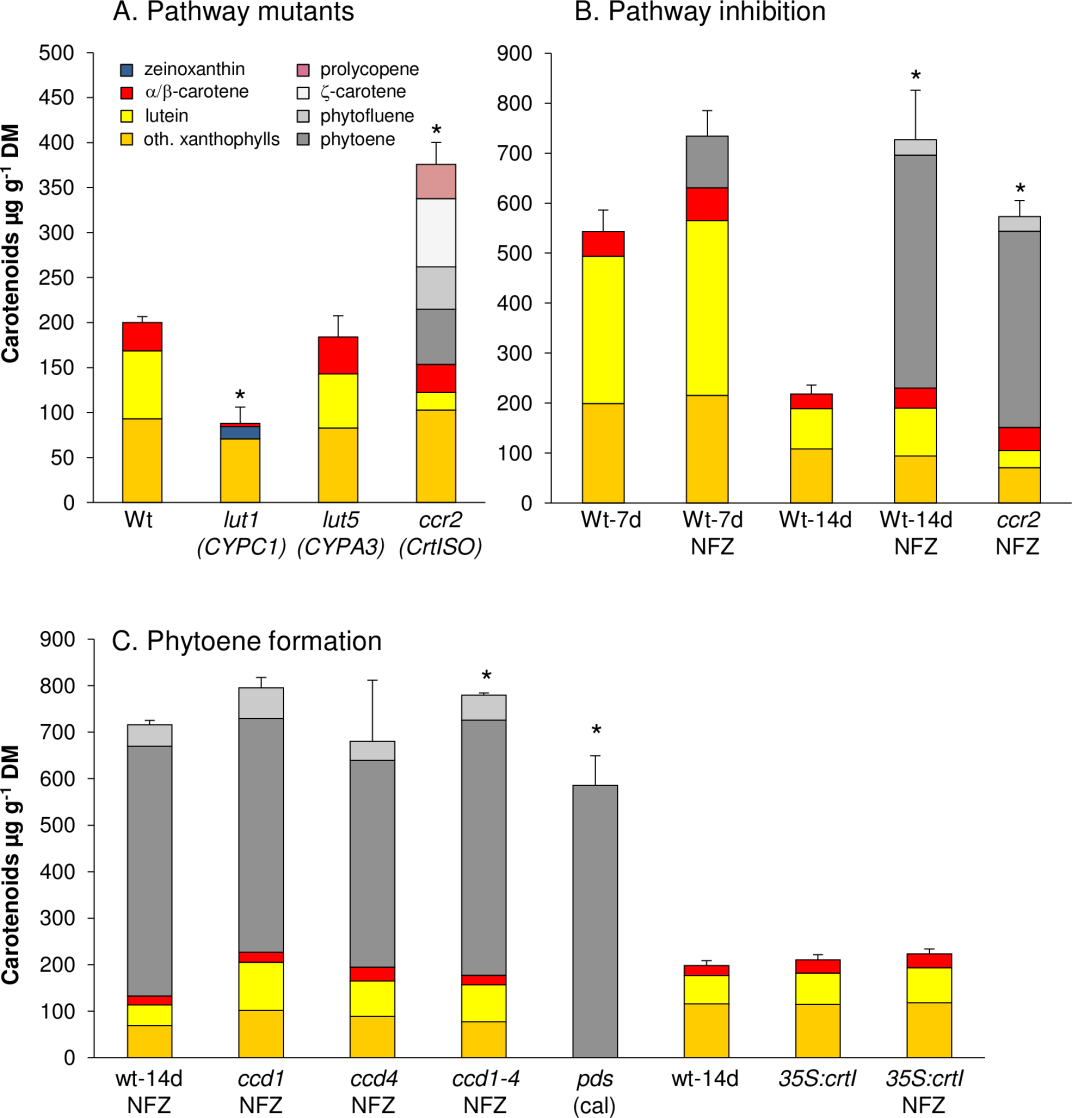
Carotenoid contents in carotenoid pathway mutants calli. A, Seed-derived calli were generated from Arabidopsis carotenoid pathway mutants. Only the *crtISO* mutant *ccr2* accumulated high levels of non-coloured pathway intermediates, while carotenoids in other mutants are almost similarly degraded. Genes affected by the mutation are given below mutant names (see Fig 1). B, Active carotenogenesis during etiolated callus development evident by phytoene accumulation in WT callus generated in presence of the PDS inhibitor norflurazon (NFZ) for 7 and 14 days. C, Similar phytoene levels in NFZ-treated calli from *ccd1* and *ccd4* excludes phytoene as *in vivo* CCD substrate. Callus from homozygous *pds* mutant accumulate phytoene in absence of NFZ while expression of the bacterial desaturase CrtI (*35S*:*crtI*) bypasses NFZ-inhibition. Results are mean ± SD from three biological replicates. Significant difference (*P*<0.05) *rel. to WT, 14d (A, B); *rel. to WT-NFZ, 14d (C). Carotenoid differences between WT and *35S*:*CrtI* calli were non-significant (*P*<0.05).

*Lut1* calli accumulated about 15% zeinoxanthin while lutein was absent matching with the carotenoid profile of *lut1* leaves [49]. Calli from *lut5* had slightly more α-carotene than the WT, mirroring the α-carotene abundance in *lut5* leaves (Fig 3A) [24]. However, surprisingly, *crtISO* calli accumulated almost two-fold higher total carotenoid amounts compared to WT calli. Although *crtISO* calli had less lutein than WT calli as previously reported for leaves, the increase was mostly due to about 250 μg g^-1^ of carotene desaturation intermediates. This included pro-lycopene and similar proportions of phytoene, phytofluene and ζ-carotene. The accumulation of these intermediates in (dark-grown) calli resembles their abundance in etiolated *crtISO* seedlings and corroborates that CRTISO is indispensable for carotene *cis-trans* isomerization in the dark [19,20]. Therefore, *cis*-carotene biosynthesis in etiolated calli persists and the large accumulation of *cis*-carotene intermediates indicates their reduced susceptibility towards carotenoid degradation in contrast to the downstream carotenoids.

### Carotenoid synthesis during Arabidopsis callus development

High carotene levels in *crtISO* calli might be caused by feed-forward increased PSY activity caused by apocarotenoid signals which are assumed to originate from *cis*-configured carotenes [16,52,53]. To determine the pathway flux in calli, we took advantage of the PDS inhibitor norflurazon (NFZ). NFZ-mediated phytoene accumulation is frequently correlated with PSY activity, in both etioplasts and chloroplasts [13,54,55]. For NFZ-treatment of calli, WT seedlings were light-germinated on CIM and thereafter transferred onto CIM supplemented with NFZ and maintained in the dark. Carotenoid patterns were determined after 7 and 14 days.

The accumulation of coloured carotenoids was decreased as expected, but was not significantly different in NFZ-treated versus non-treated WT calli (Fig 3B). However, NFZ treatment caused 100 and 470 μg g^-1^ of phytoene to accumulate after 7 and 14 days in the dark, respectively (S2 and S3 Figs). The equivalent experiment with *crtISO* calli led to approximately 400 μg g^-1^ phytoene after 14 days etiolation thus almost matching the amounts in WT calli. This argues against feed-forward induced pathway activation in *crtISO* calli and indicates similar pathway activities in both WT and *crtISO* during callus etiolation.

Given its continuous accumulation during callus etiolation, phytoene appears to be unaffected by degradation. As CCD1 is reported to cleave phytoene *in vitro* [56], we determined possible enzyme-mediated phytoene losses by quantification of phytoene amounts in calli from *ccd1*, *ccd4* and the *ccd1 ccd4* double mutant grown on NFZ (Fig 3C). Partial PDS inhibition by NFZ caused low amounts of phytofluene to accumulate in addition to phytoene which slightly increased only in *ccd1* calli compared to the WT. In contrast, phytoene levels in all *ccd* mutants were not significantly different from that of WT calli, thus excluding that phytoene is an *in vivo* substrate for CCD1 and CCD4-catalyzed cleavage.

Moreover, we included calli from an Arabidopsis *pds* mutant, thus representing a genetic equivalent to NFZ-inhibition [57]. As homozygous *pds* mutants develop only bleached cotyledons, but then cease further development, we generated calli from cotyledons. In agreement with a constant pathway activity during callus development, *pds* calli accumulated similar phytoene levels like NFZ-treated WT calli (Fig 3C). These interrelations were further confirmed with calli from Arabidopsis lines expressing the bacterial phytoene desaturase *CrtI* which bypasses PDS-mediated phytoene desaturation thus conferring insensitivity to NFZ [58,59]. As expected, *35S*::*CrtI* calli accumulated carotenoid amounts similar to WT and did not accumulate phytoene in presence of NFZ.

### High phytoene synthesis rates exceed carotenoid breakdown and result in β-carotene accumulation

Clearly, phytoene is not subjected to degradation in calli and there does not appear to be a feed-forward effect of phytoene accumulation on carotenoid biosynthesis. Thus, phytoene levels accumulating upon NFZ treatment can be used as a measure for monitoring carotenoid pathway activity during callus etiolation undiminished by enzymatic and non-enzymatic degradation. In addition to those carotenoids present in light-grown callus prior to etiolation (1.5 mg g^-1^), most of the carotenoids synthesized during callus etiolation (0.5 mg g^-1^, as phytoene) are promptly degraded. Quantitatively, a total amount of 2 mg g^-1^ carotenoids are degraded within 14 days of etiolated callus development.

**Fig 4:**
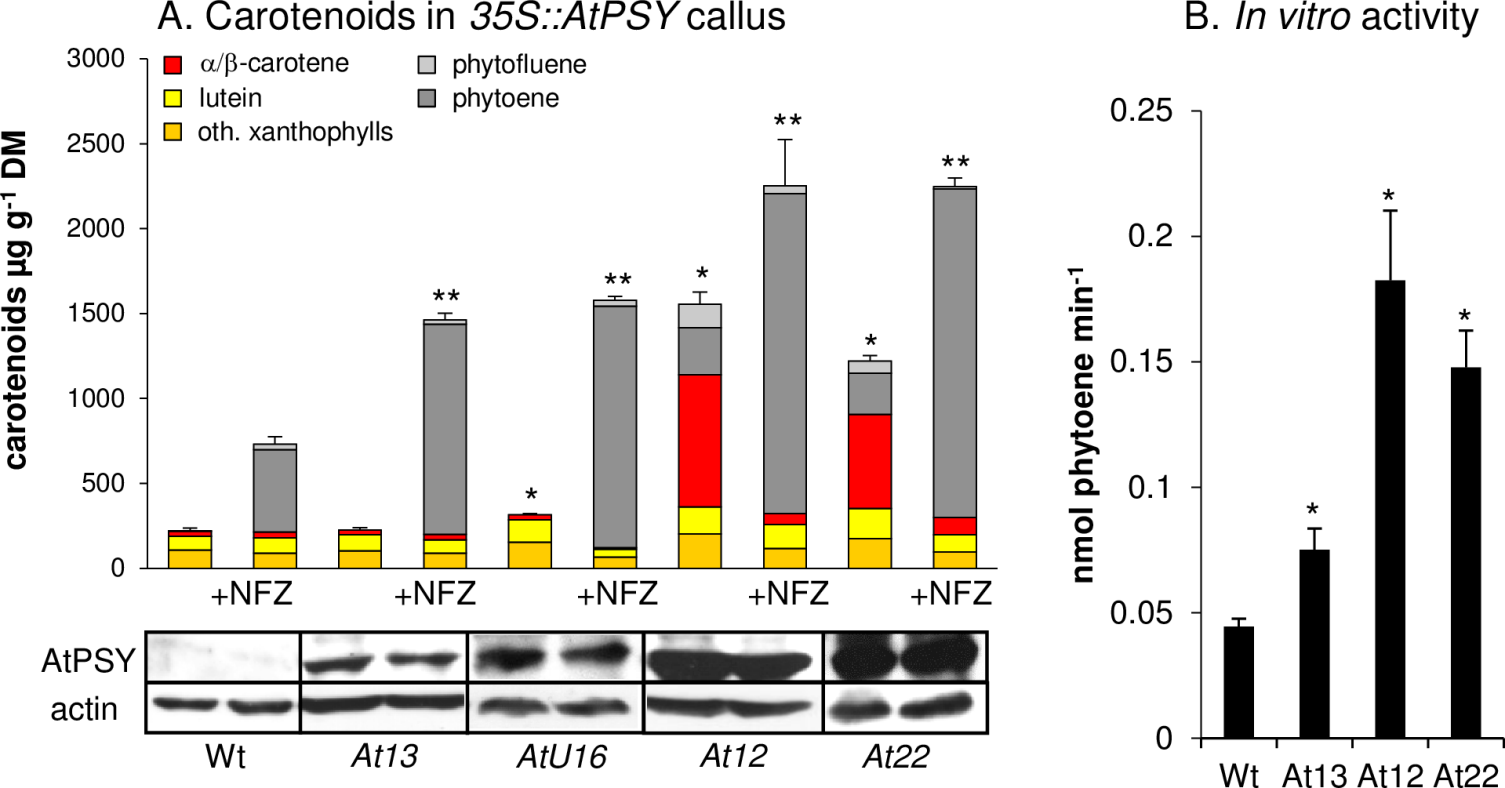
Carotenoid pathway flux determines β-carotene accumulation in Arabidopsis callus. A, Seed-derived calli were generated from *AtPSY*-overexpressing Arabidopsis lines with low (*At13*, *AtU16*) and high (*At12*, *At22*) PSY protein levels. *At13* and *AtU16* accumulated carotenoid levels almost like WT despite two-fold increased pathway activity concluded from the phytoene content in presence of NFZ. Further increased pathway activity as in lines *At12* and *At22* results in β-carotene accumulation. Different PSY activities are reflected by different PSY protein levels shown by immunoblotting using 80 μg of callus protein below. Actin levels are included as loading control. Results are mean ± SD from at least three biological replicates. Significant difference to the WT (*) and WT-NFZ (**), respectively, *P*<0.05. B, *In vitro* PSY activities were determined in isolated callus plastid membrane fractions by incubation with DMAPP, [^14^C]-IPP and a recombinant GGPP synthase and quantification of [^14^C]-phytoene levels. Results are means ± SD from three biological replicates. Significant difference to WT, *P<0.05.

The pronounced carotenoid degradation capacity conflicts with high carotenoid amounts usually achieved through *PSY* overexpression in various tissues, including Arabidopsis calli as previously demonstrated [44,60]. In calli from two selected *AtPSY*-overexpressing lines (*At12* and *At22*), there was a significant increase in xanthophylls especially lutein (2-3-fold) and α/β-carotene (20-30-fold) and a substantial accumulation of phytoene (240–280 μg g^-1^) as well as phytofluene (73–137 μg g^-1^) (Fig 4; S4 Fig for calli images). Given the strong resistance of phytoene and phytofluene against degradation as described above (Fig 3), the accumulation of these carotenes is not surprising. However, high amounts of β-carotene are unexpected as β-carotene is reduced by 90% during WT callus development (Fig 2A). High levels of β-carotene together with colourless intermediates is a widespread pattern for *PSY*-overexpressing tissues, and is similarly observed for *PSY*-overexpressing maize and rice calli [61,62].

Interestingly, in a screen of calli from about 50 additional *AtPSY*-overexpressing Arabidopsis lines, we noticed that calli either turned orange or remained as pale as WT calli while intermediate colour intensities were not observed. This was confirmed by HPLC analysis of calli from two selected pale lines (*At13*, *AtU16*) which revealed carotenoid levels and patterns just like the WT, while colourless pathway intermediates were also absent (Fig 4A). Compared with the two *35S*::*AtPSY* lines forming orange calli (*At12*, *At22*), the paler lines had much lower PSY protein levels suggesting that the carotenogenic pathway activity determines whether β-carotene over-accumulates or not (Fig 4A).

To corroborate this, we determined pathway activity using *PSY* overexpression lines by NFZ inhibition (Fig 4A). Pale calli from lines *At13* and *AtU16* accumulated about 1500 μg g^-1^ phytoene—thus about three-fold more than WT calli. However, NFZ-treated calli from orange lines (*At12*, *At22*) further exceeded these levels by accumulating almost 1900 μg g^-1^ phytoene. These different pathway capacities were confirmed by *in vitro* PSY activity assay using callus plastid membrane fractions (Fig 4B). Upon incubation with DMAPP, [^14^C]-IPP and a recombinant Arabidopsis GGPP synthase, plastids from the pale-callus line *At13* formed about 75% higher [^14^C]-phytoene levels than WT, while orange-callus lines formed about 300% higher [^14^C]-phytoene levels.

In conclusion, a moderately-enhanced carotenoid pathway activity through *PSY* overexpression does not necessarily result in carotenoid over-accumulation in dark-grown calli. Considering our findings on carotenoid degradation, moderately increased carotenoid activity appears to be promptly compensated by degradation as in weaker *PSY-*overexpression lines *At13* and *AtU16*. However, stronger pathway activity by stronger *PSY* overexpression (*At12*, *At22*) produces carotenoid levels which exceed the breakdown rate and result in increased steady-state levels of β-carotene and upstream intermediates. The upstream carotene intermediates like phytoene and phytofluene most probably accumulate as a consequence from the oversaturation of desaturation capacities (see Discussion).

### Carotenoid degradation products in Arabidopsis calli

**Fig 5:**
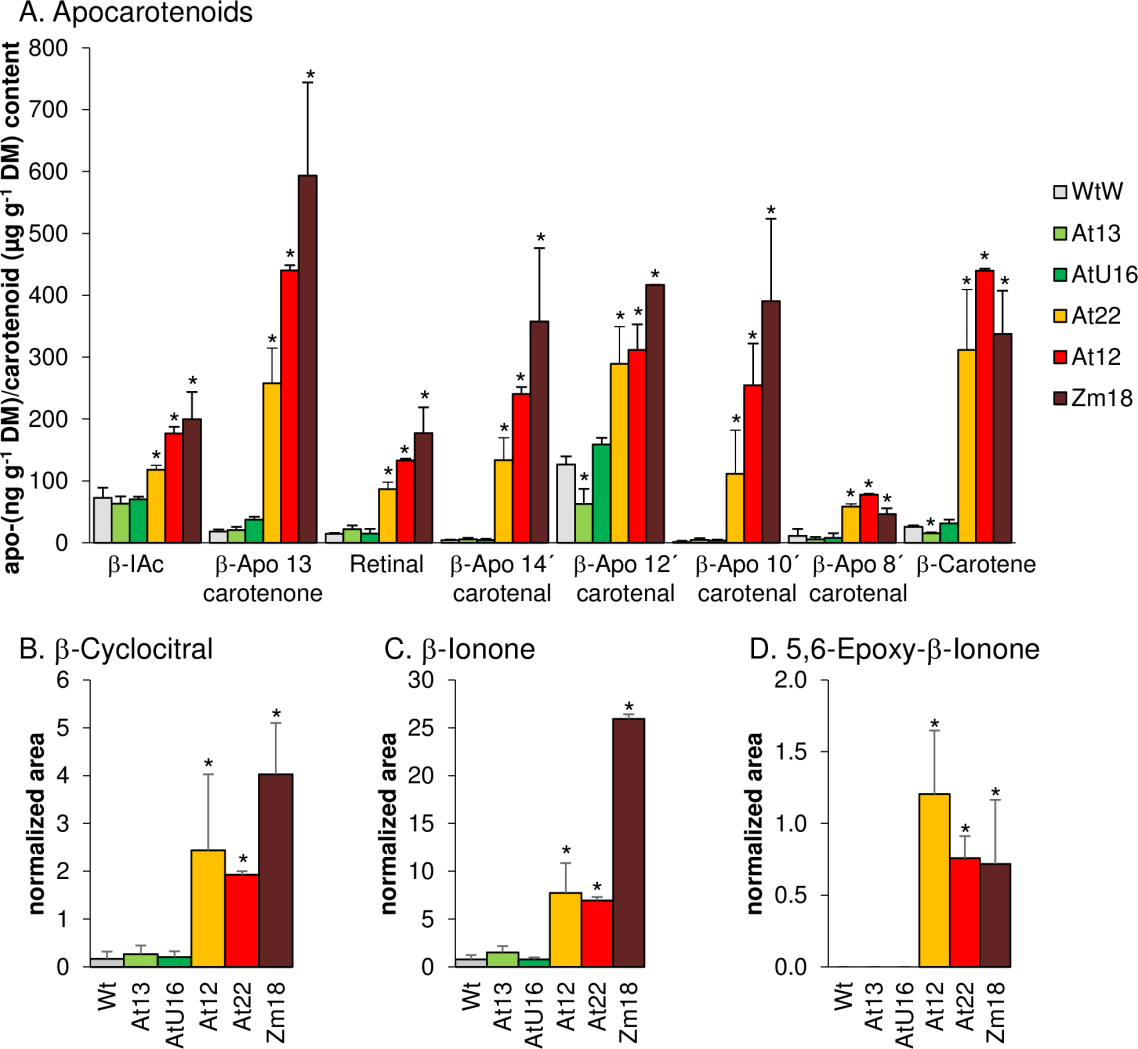
β-Apocarotenoids in Arabidopsis callus. A, β-Apocarotenals were determined in seed-derived calli from *AtPSY*-overexpressing (*At12*, *At22*) and *ZmPSY1*-expressing (*Zm18*) Arabidopsis lines by LC-MS analyses. Apocarotenoid amounts were expressed in ng g^-1^ DM. β-IAc, β-ionylidene acetaldehyd. The amounts of β-carotene (in μg g^-1^ DM) are given for comparison. B, β-ionone, C, β-cyclocitral and D, 5,6-epoxy-β-ionone are expressed as peak areas normalized to internal standards and dry mass. Results are means ± SD from three biological replicates. Significant difference to the WT, *P<0.05.

Despite increased phytoene synthesis the pale/weaker *AtPSY*-overexpressing calli did not accumulate coloured carotenoids which may indicate the prevalence of increased carotenoid breakdown. In leaves, increased pathway activity is compensated by higher levels of glycosylated xanthophyll-derived apocarotenoids, as we showed recently [13]. In contrast, roots were shown to accumulate β-apocarotenals instead of apocarotenoid glucosides. Given the similarity of calli to meristematic root cells, we only found increased levels of β-apocarotenals but no glucosides, as expected (Fig 5A). We identified β-apocarotenals of various chain lengths at low levels in WT calli, including retinal, β-apo-14’-carotenal, β-apo-12’-carotenal, β-apo-10’-carotenal, β-apo-8’-carotenal, β-apo-13-carotenone and β-ionylidene acetaldehyde. All β-apocarotenals were more abundant in β-carotene over-accumulating calli, while they remained similar to the WT in pale calli from the weaker *AtPSY-*overexpression lines. Therefore, β-apocarotenal levels correlated well with increased β-carotene levels, but not necessarily with increased pathway activity. Moreover, we identified the volatiles β-cyclocitral, β-ionone and 5,6-epoxy-β-ionone, which similarly showed higher abundances exclusively in lines over-accumulating β-carotene (Fig 5B–5D). These results were further confirmed using calli from one *ZmPSY1*-expressing line, which over-accumulates β-carotene just like *AtPSY*-overexpressing lines [60] and showed similar increases in apocarotenoid levels (Fig 5). Moreover, we determined about 50-fold increased amounts of geronic acid, which is liberated from carotenoid-oxygen copolymers and corroborates the non-enzymatic degradation of β-carotene in calli (S5 Fig) [38,63].

### Kinetics of β-carotene formation and decomposition in calli

**Fig 6:**
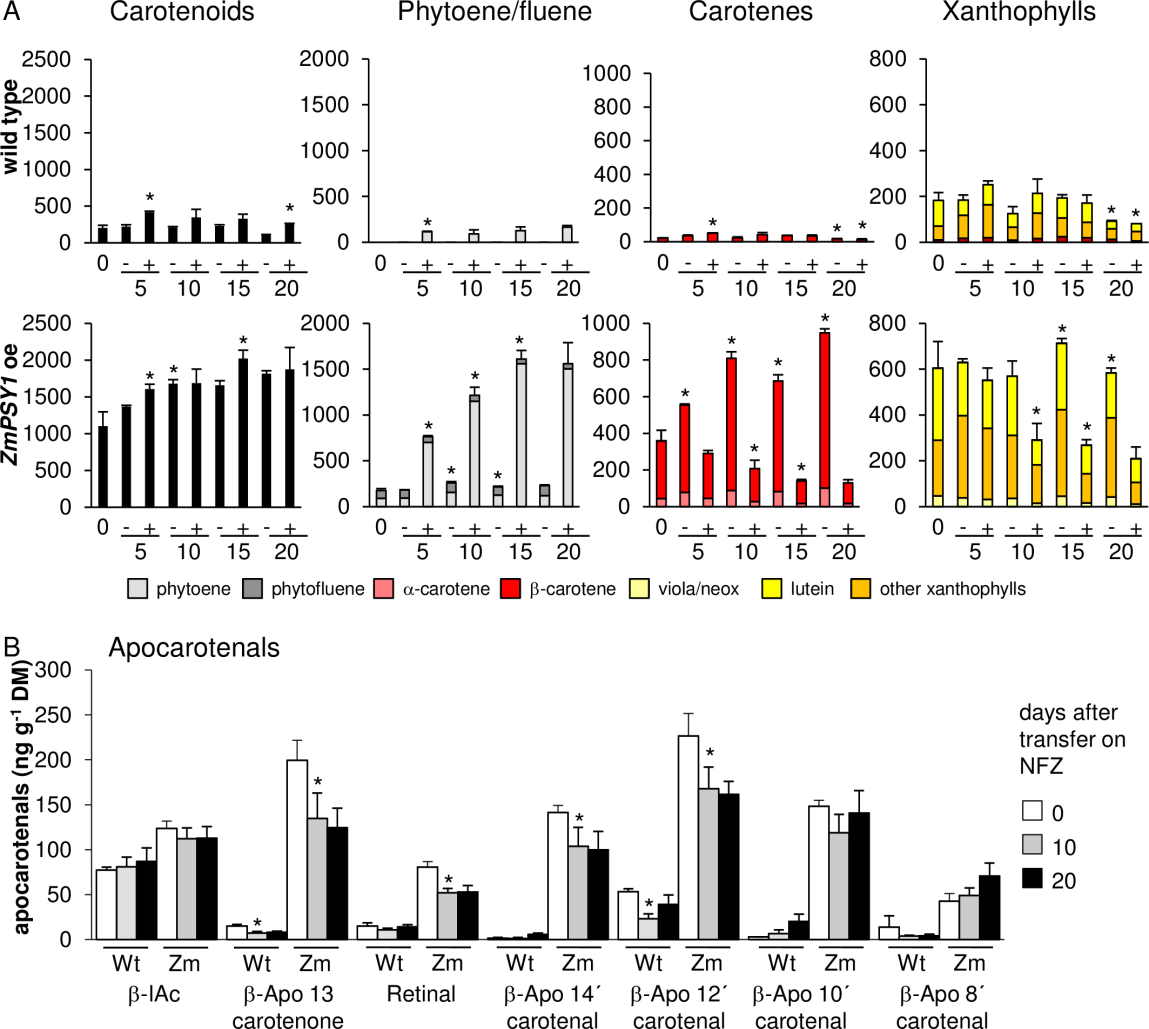
Carotenoid kinetics during prolonged callus development. WT and *ZmPSY1*-expressing seeds (Zm) were germinated for 5 days on callus-inducing medium (CIM) under long day, followed by 2 weeks etiolation. Thereafter, calli were transferred on CIM (-) or CIM supplemented with 1 μM NFZ (+) and developed further in the dark. Samples were taken after 0, 5, 10, 15 and 20 days and carotenoids were quantified by HPLC (A) while β-apocarotenals were quantified at 0 days and after 10 and 20 continued etiolation on NFZ by LC-MS (B). Note that carotenoids are given in μg g^-1^ DM, while β-apocarotenals are in ng g^-1^ DM. All data are mean +/- SD from three biological replicates. Significant difference to preceding time point of same sample type, *P<0.05).

Taking advantage of the possibility to arrest β-carotene synthesis, we investigated β-carotene stability after treatment with NFZ. For this purpose calli from WT and the *ZmPSY1*-expressing line were etiolated for 14 days and thereafter transferred onto CIM supplemented with 1 μM NFZ and carotenoids quantified after 5, 10, 15 and 20 days continued etiolation. In addition, prior to inhibitor treatment and after 10 and 20 days inhibition, β-apocarotenoids were quantified. Non-treated control calli were grown accordingly and transferred onto fresh CIM medium without NFZ (Fig 6).

In non-inhibited control calli, carotenes and xanthophylls remained relatively constant over time in the WT and increased only slightly in the *ZmPSY1*-expressing line. Upon NFZ inhibition phytoene levels increased constantly in both WT and *ZmPSY1*-expressing calli, while visible carotenoids decreased concurrently due to ongoing oxidative degradation (Fig 6A). The decrease in visible carotenoids occurred almost linearly within the initial 15 days following pathway inhibition and then remained almost constant possibly due to nutrient shortage. While most β-apocarotenal levels remained constant with prolonged development in NFZ-treated WT calli, β-apo-13-carotenone, retinal, β-apo-14’-carotenal and β-apo-12’-carotenal in NFZ-inhibited *ZmPSY1*-expressing calli were reduced by approximately 30% after 10 days etiolation and remained constant after 20 days etiolation (Fig 6B).

Quantitative comparisons of carotenoid synthesis and degradation rates allowed calculating the pathway flux required for the net accumulation of β-carotene. In *ZmPSY1*-expressing calli the phytoene increases in presence of NFZ reflects an average daily synthesis of about 180 nmol (100 μg g^-1^). In non-inhibited calli, β-carotene increased with only about 50 nmol (27 μg g^-1^) per day, while xanthophylls remained relatively constant. Therefore, about 130 nmol (70 μg g^-1^) β-carotene per day was either converted into xanthophylls (and immediately subjected to further degradation) or oxidatively degraded. Accordingly, this suggests that synthesis rates exceeding 130 nmol (70 μg g^-1^) β-carotene per day are required to overcome the turnover rate and to over-accumulate β-carotene. Interestingly, this matches with the different effect on carotenoid over-accumulation observed with *AtPSY-*overexpressing calli from above (Fig 4). Pale lines (*At13* and *AtU16*) had an average daily synthesis of 100 to 123 nmol respectively, which correspond with the pathway activity compensated by degradation as described above. In contrast, carotenoid over-accumulating lines synthesized approximately 190 nmol of carotenoids per day, which exceeds the degradation rate and thus explains the net over-accumulation of carotenoids.

In order to correlate β-apocarotenal levels with β-carotene losses on a molar basis, we accounted for the fact that β-carotene forms cleavage product pairs (β-apo-14’-carotenal / β-apo-13-carotenone; retinal / retinal etc.). β-Apocarotenal levels in *ZmPSY1-*expressing calli correspond to 41 nmol β-carotene degraded prior to prolonged callus incubation and dropped to 30 nmol after 10 days of pathway inhibition. However, since 50 nmol β-carotene is degraded per day, β-apocarotenals are evidently in steady state and are also likely to be continuously degraded into further yet unknown metabolites.

### Secondary β-apocarotenal oxidation products

**Fig 7:**
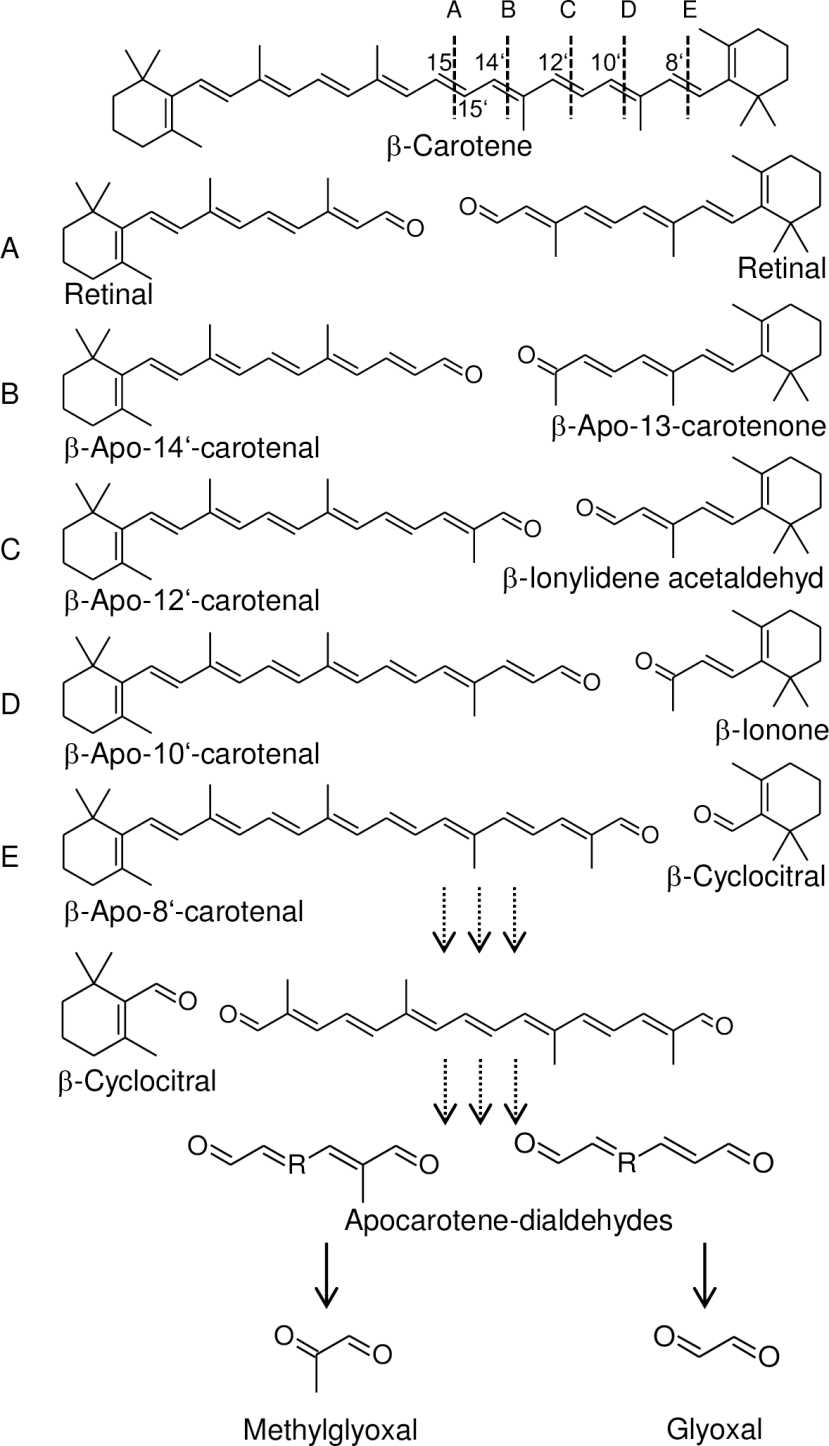
β-Carotene oxidation products. β-Carotene oxidation primarily yields β-apocarotenals of various chain lengths. Capital letters indicate the reacting double bond position; the corresponding cleavage product pairs are depicted below from A to E. Secondary oxidation results in the release of the β-ionone ring moiety, e.g. as β-cyclocitral, and linear apocarotene-dialdehydes. Methylglyoxal and glyoxal represent end products after continued oxidation of carotene dialdehydes.

**Fig 8:**
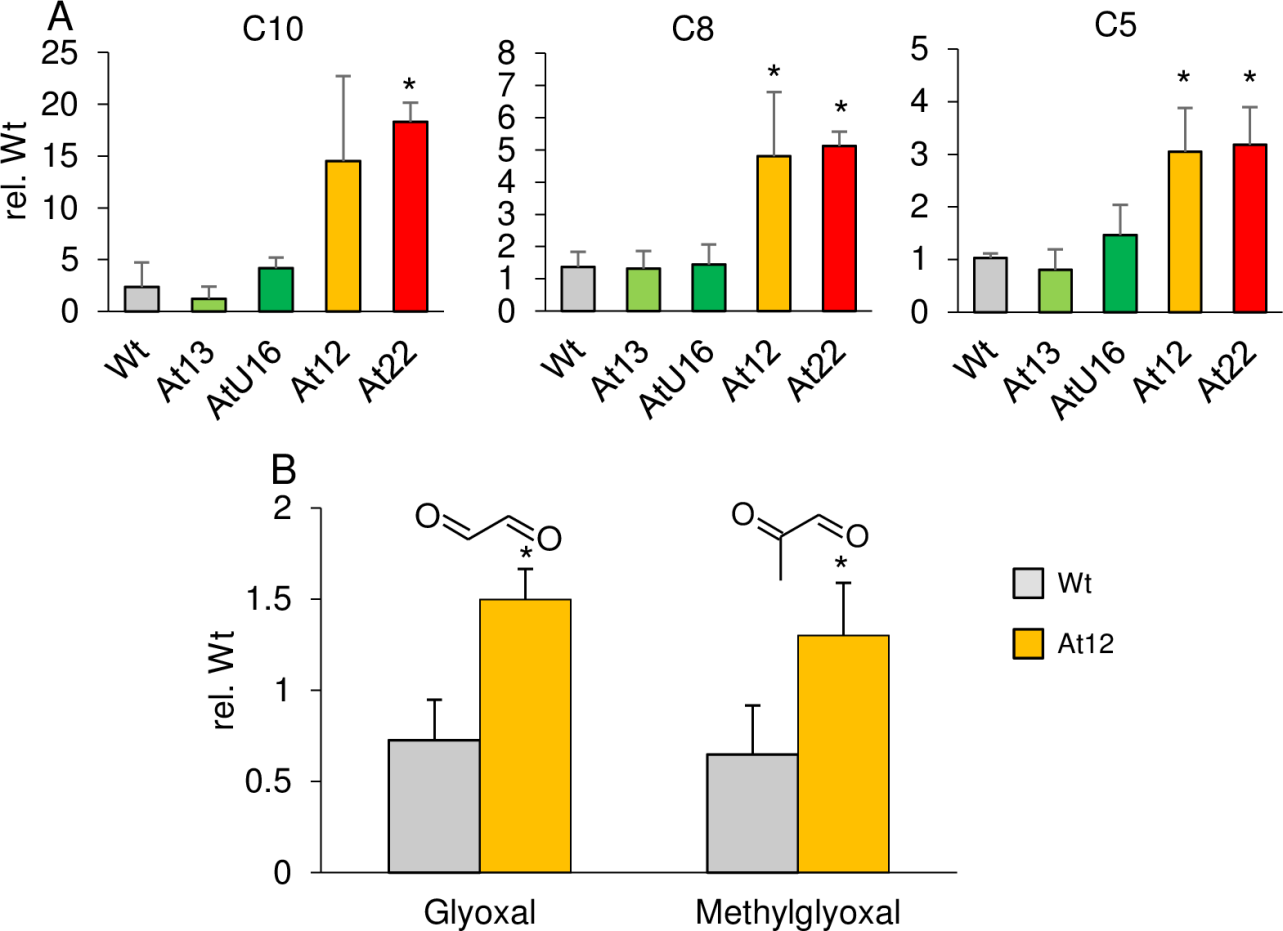
Apocarotene-dialdehydes in Arabidopsis callus. Apocarotene-dialdehydes were determined in seed-derived calli from *AtPSY*-overexpressing Arabidopsis lines by LC-MS analyses. Peak areas were normalized to internal standards and dry mass and are expressed relative to one WT sample. Data are mean ± SD from three biological replicates, significant difference rel. to WT (*P<0.05). Numbers indicate dialdehyde hydrocarbon chain length, ranging from C10 to C5 (A); relative glyoxal and methylglyoxal levels were determined for WT and *At12* calli and are shown in B. For detailed compound names, see S2 Table.

The presence of β-apocarotenals with various chain lengths suggests non-selective oxidation at any double bond present in β-carotene. Continued oxidative cleavage of long-chain β-apocarotenals might yield short-chain β-apocarotenals (β-cyclocitral, β-ionone) plus dialdehydes of various chain lengths (Fig 7). We analyzed calli from *AtPSY*-overexpressing lines with different pathway activities for corresponding apocarotene-dialdehydes by LC-MS (Fig 8A). In fact, we identified apocarotene-dialdehydes with variable chain lengths ranging from C10 to C5 while other apocarotene-dialdehydes were below detection limit. Relative quantification of these apocarotene-dialdehydes revealed increased levels compared to WT calli only in correlation with high β-carotene and β-apocarotenal levels as in orange-coloured calli from *AtPSY*-overexpressing lines. Moreover, continued subsequent oxidative truncations of apocarotene-dialdehydes might result in glyoxal and methylglyoxal as shortest possible dialdehydes (Fig 7). Confirmatory, we found about 50% increased levels of both dialdehydes in calli of *AtPSY*-overexpressing line *At12*, compared to WT calli (Fig 8B).

### Subcellular localization of carotenoid crystals

We previously demonstrated that β-carotene in *PSY*-overexpressing lines over-accumulates as crystals in Arabidopsis calli, similar to carrot roots [44]. The plastid-localization of carotenoid biosynthesis indicates that these crystals are present within these organelles. However, this would imply drastic changes in plastid morphology, as crystals observed are much larger compared to plastids present in control cells and are needle- or rhombus-shaped. Moreover, Cao et al. observed carotenoid crystals in *Citrus* calli in amyloplasts as well as within vacuoles [64].

**Fig 9:**
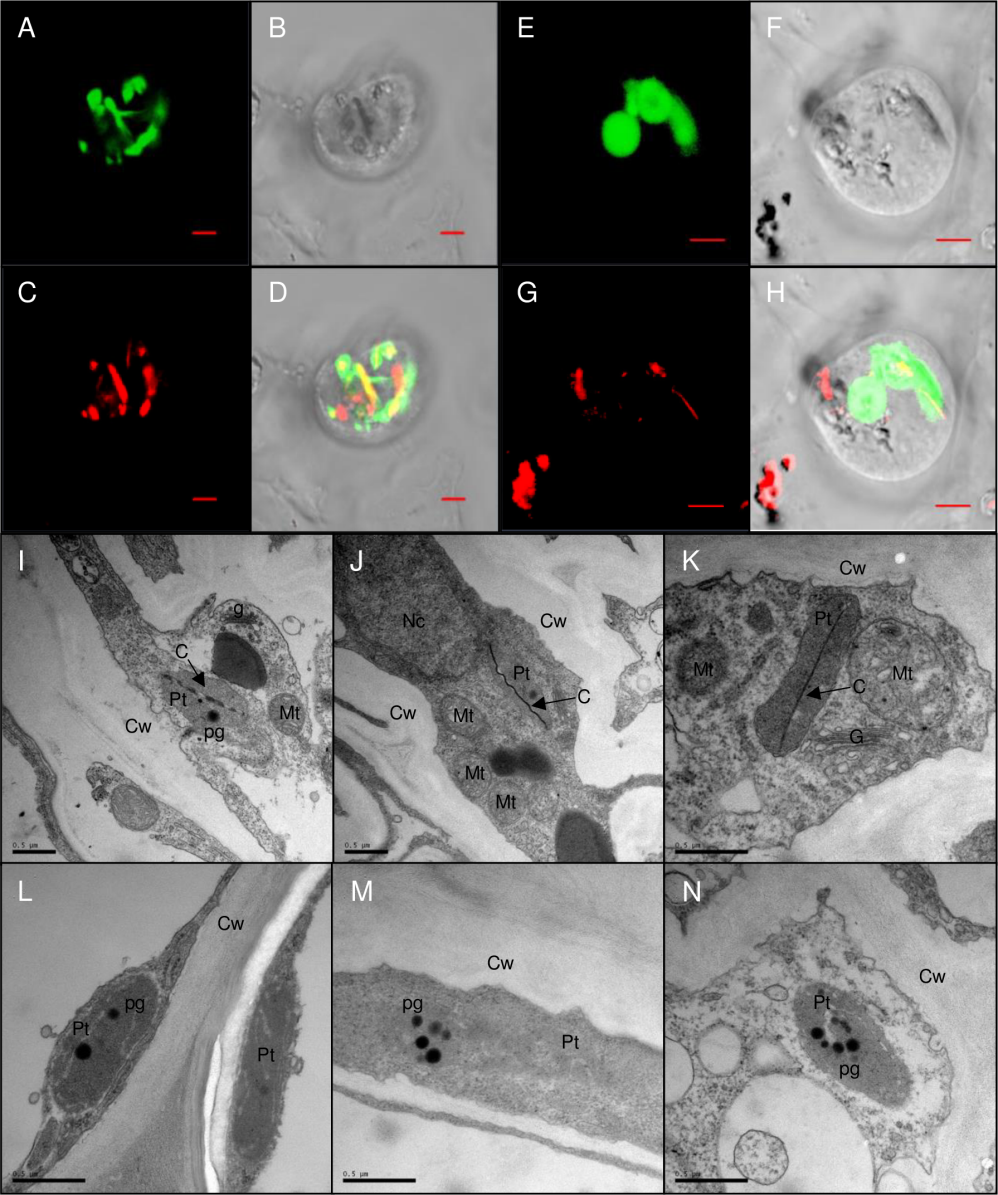
Carotenoid crystal formation in Arabidopsis. A to H, one *AtPSY*-overexpressing line was crossed with an Arabidopsis line expressing a plastidic marker protein fused to cyan fluorescent protein (CFP). Two different callus protoplasts are shown (A to D and E to H). CFP fluorescence (A, E) largely overlaps with carotenoid crystals visualized using a (polarized) laser beam at 543 nm (C, G), confirming intraplastid localization of carotenoid crystals. B and F, bright field images; D and H, overlay of all images. Slight mismatches between CFP and crystal birefringence are due to plastid movements between two image acquisitions. Bar = 5 μm. I to N, TEM of roots sections of an *AtPSY*-overexpressing line (I to K), and roots of Arabidopsis wild type (L to N). Cw, cell wall; Pt, plastid; Mt, mitochondrium; Nc, nucleus; pg, plastoglobuli; G, Golgi; C, membrane remnants of carotenoid crystal. Bar = 0.1 μm.

To address these questions we sought for non-invasive localization of crystals in orange *AtPSY*-overexpressing Arabidopsis calli. We crossed line *At22* with an Arabidopsis line expressing a plastid-marker protein fused to cyan fluorescent protein (CFP) [65]. Protoplasts were isolated from calli and analyzed by fluorescence microscopy. Carotenoid crystals were visualized with a (polarized) laser beam while plastids were identified through CFP fluorescence (Fig 9A to 9H). The spatial match of both signals unequivocally confirmed the intra-plastidic localization of crystals. Moreover, we analyzed plastid structures from a crystal-forming *AtPSY*-overexpressing line by transmission electron microscopy. For this, we used roots which were recently shown to accumulate similarly high carotenoid levels like callus [44]. This revealed plastid-localized membrane remnants exclusively in the carotenoid-accumulating line, while these structures were absent in WT roots (Fig 9I to 9N). As most lipophilic carotenoids are extracted during sample dehydration, undulating membranes which formerly surrounded carotenoids are indicative for crystals and are similarly found in tomato and carrot chromoplasts [66,67].

## Discussion

### Arabidopsis callus as model system for quantifying carotenoid pathway flux

Nutritionally important carotenoids often accumulate in non-green tissues of crops, such as fruits, seeds and storage roots [10] with carotenoid-sequestering protein complexes and structures contributing essentially to carotenoid stability. While chromoplasts form specialized storage structures, chloroplasts sequester carotenoids mainly within membranes and protein-bound in light-harvesting complex proteins [68]. Although Arabidopsis serves as model for multifaceted plant research, unravelling the fundamental processes governing carotenoid stability and deposition has been hampered due to the absence of chromoplasts. The establishment of the non-green callus system allows carotenoid biosynthesis and degradation to be mechanistically investigated in Arabidopsis. This assay system can likely assist in developing strategies to develop improved provitamin A-biofortified crops.

We here demonstrate large differences of carotenoid species regarding their degradation. Carotenes bearing cyclic end groups like α- and β-carotene were degradation-sensitive and decreased rapidly upon callus etiolation or when biosynthesis was blocked using NFZ (Figs 2 and 6). However, carotenes with cyclic end-groups were maintained at higher steady-state levels when carotenoid pathway activity was increased through overexpression of *PSY*. Various crop tissues show chemotypic similarities with Arabidopsis calli, sharing similar carotenoid patterns. The tomato *ghost* mutant for instance over-accumulates phytoene due to an impaired phytoene desaturation [69–71]. Interestingly, total carotenoid amounts in *ghost* fruits are almost doubled compared to red-ripe WT tomatoes. This suggests significant carotenoid losses when the pathway is unrestricted, like in WT tomatoes, i.e. when phytoene is further metabolized into downstream carotenoids. These losses are partially due to enzymatic cleavage of downstream carotenoids into aromatic volatiles, but a large fraction is expected to be degraded non-enzymatically [32,72,73]. In line with this, we observed high phytoene amounts in NFZ-treated WT calli which—in absence of pathway inhibition–would be carried into carotenoids with cyclic end-groups and subsequently degraded resulting in much lower total carotenoid levels.

While phytoene is the predominant carotene accumulating upon NFZ inhibition, downstream carotenes like phytofluene, ζ-carotene and pro-lycopene are similarly degradation-resistant. These carotenes accumulated stably during callus etiolation when the pathway was blocked chemically or by the loss of key pathway enzymes such as PDS or CRTISO (Fig 3). Equivalently, *crtISO* mutants of tomato (*tangerine*) and melon (*yofi*) accumulate pro-lycopene and upstream carotenes to larger amounts compared to total carotenoids in corresponding WT fruits with unrestricted pathway flux [19,74,75].

Finally, the seed-derived callus assay allowed us to enhance pathway activity through the overexpression of *PSY*, which revealed carotenoid patterns matching those observed in carotenoid-rich, orange-coloured crop tissues (Fig 4). For instance, β- and α-carotene are the main carotenes followed by colourless intermediates in carrot roots, sweet potatoes and squash [44,50,76,77]. The specific accumulation of β-carotene upon high pathway activities reflects a limited β-carotene hydroxylation capacity while additional phytoene and phytofluene accumulation is caused by limited desaturation capacity and maintained by their resistance to oxidative cleavage. These interrelations are convincingly evident from successful approaches to increase β-carotene levels through down-regulation of hydroxylases [78–80]. Overall, our seed-derived callus assay system appears useful for an exploration of carotenoid homeostasis involving biosynthesis and degradation.

### Different susceptibilities of carotenoid species to non-enzymatic degradation

Enzyme-mediated carotenoid cleavage generates various aroma compounds and is mediated by CCD enzymes [72,81]. With the exception of mutants deficient in CCD1 and CCD4, other carotenoid cleavage enzymes did not contribute to major carotenoid turnover as corresponding mutants showed unaltered carotenoid breakdown in calli (Fig 2). Increased carotenoid levels in *ccd1* and *ccd4* calli are in accordance with higher carotenoid levels reported for dark-incubated *ccd1* and *ccd4* Arabidopsis leaves and corroborates the contribution of these CCDs to carotenoid turnover [36]. However, carotenoid cleavage enzymes are probably not involved in β-apocarotenal formation generated upon β-carotene over-accumulation in *PSY*-overexpressing calli. While β-apocarotenals of all possible chain lengths were detected, CCD1 and CCD4 are known to selectively cleave C7-C8 and C9-C10 double bonds (Fig 5) [82,83]. Moreover, callus generation in presence of a vitamin E analog retarded carotenoid losses suggesting that carotenoids are primarily degraded non-enzymatically, thus through oxidation.

The conjugated polyene is the site of oxidation. While β-carotene has 11 conjugated double bonds, even those species with small chromophores, like phytoene and phytofluene with three and five conjugated double bonds, respectively, would be expected to be prone to oxidative attack. In contrast to prominent carotenoids like lycopene and β-carotene, the antioxidative capacity of phytoene and phytofluene was assessed only recently [84–86]. In fact, purified phytoene and phytofluene in solution were susceptible to autoxidation [87,88] and recent analyses confirmed their antioxidant activities, although weaker compared with lycopene and β-carotene [89]. Furthermore, lycopene isomers including pro-lycopene had the same antioxidant capacity like all-*trans*-lycopene and α-tocopherol [90].

However, these results disagree with our observations. Linear carotenes from phytoene to pro-lycopene were highly resistant against oxidative destruction as they accumulate in calli (Fig 3). Possibly, these carotenes are less accessible for oxygen *in vivo*, which might be caused by different sequestration modes compared to β-carotene. This agrees with fractionation experiments carried out with tomato chromoplasts equipped with different carotenoid patterns [91]. While phytoene was sequestered within plastoglobules all-*trans*-lycopene and β-carotene were found in thylakoid-like membranes and formed crystals, apparently due to a limited membrane capacity to harbour these carotenes. Moreover, the sequestration modes of lycopene isomers differ dramatically: while pro-lycopene formed liquid-crystalline structures in CRTISO-deficient *tangerine* tomatoes, all-*trans*-lycopene in WT fruits formed crystals [92]. Given the crucial impact of food matrix on bioaccessibility, the liquid-crystalline sequestration of phytoene, phytofluene and pro-lycopene might account for their higher bio-accessibility compared with other carotenoids [10,86,92]. Nonetheless, our seed-derived callus assay corroborates that *cis*-carotenes are less susceptible to oxidative cleavage, highlighting them as rather stable intermediates which might also favour them as substrates for several proposed apocarotenoid signals [16].

### Oxidative cleavage of carotenoids during callus development

In photosynthetic complexes, carotenoids are bound to light-harvesting complex proteins. Calli developed from light-grown tissues lose most of their carotenoids during etiolation. The decomposition of photosynthetic apparatus most probably releases carotenoids increasing oxidative susceptibility, resembling senescence-mediated catabolism in chloroplasts. In fact, decomposition of thylakoid membranes during leaf senescence is accompanied by carotenoid degradation which is thought to involve plastoglobuli [93]. Interestingly, etiolated calli accumulated xanthophyll esters, which are formed during leaf senescence or fruit ripening and might thus represent carotenoid degradation intermediates [93,94]. This suggests that carotenoid breakdown in calli follows senescence-related pathways [95].

Carotenoids are known to be oxidized rapidly in presence of singlet oxygen present at high concentrations in photosynthetically active leaves. Confirmatory, high levels of β-apocarotenals are formed in green vegetables and also Arabidopsis leaves, initiated by non-enzymatic quenching of singlet oxygen [13,38,40,96]. Therefore, carotenoid degradation during early callus etiolation might be caused by triplet-excited chlorophylls. However, carotenoid degradation continues even in absence of chlorophylls. Interestingly, singlet oxygen is not only generated during photosynthesis—it also forms through various types of stresses and accordingly is present in low amounts in chlorophyll-free tissues. These levels might be sufficient to initiate continuous carotenoid oxidation in etiolating calli [97].

In addition to the direct oxidation of carotenoids, there is also an indirect route via lipid peroxidation products. Lipid hydroperoxides are generated non-enzymatically via reactions with singlet oxygen and radials or enzymatically by incorporation of molecular oxygen catalyzed by lipoxygenases (LOX, [98]). Secondary reactions of lipid hydroperoxides generate various radicals which attack carotenoids–especially if in close proximity which applies for membrane-embedded carotenoids. Several findings support an inverse correlation between LOX activity and carotenoid retention. For instance, a deletion at a *LOX* locus was associated with improved pasta color in durum wheat [99]. Moreover, the *r9-LOX1* gene in rice was found to be responsible for rice seed quality deterioration and *r9-LOX* RNAi Golden Rice 1 lines showed significantly increased endosperm carotenoid stability during storage [100,101].

Antioxidants like tocopherols are capable to quench both singlet oxygen as well as lipid peroxyl radicals and apparently reduce the oxidative degradation of carotenoids. This is concluded from increased callus carotenoid levels in presence of a tocopherol analogue and corroborated by other observations. For instance, α-tocopherol is positively correlated with α- and β-carotene in carrots and higher α-tocopherol levels protect lycopene and β-carotene against LOX-catalyzed co-oxidation *in vitro* [47,102]. Furthermore, elevated vitamin E levels extend β-carotene half-life during storage in sorghum [48].

### The fate of β-carotene upon oxidation

The antioxidative function of carotenoids primarily generates β-apocarotenoids which maintain their antioxidative properties [103]. Continued oxidation yields shorter apocarotene-dialdehydes of various chain lengths among which the shortest dialdehydes methylglyoxal and glyoxal cannot be oxidized further and represent possible terminal products of carotenoid oxidation (Fig 7). Confirmatory, β-apocarotenals, apocarotene-dialdehydes, glyoxal and methylglyoxal are oxidatively generated from β-carotene after treatment with ozone *in vitro* [104]. Interestingly, we found increased levels of these metabolites in correlation with high β-carotene amounts which confirms that oxidative β-carotene decomposition occurs also *in vivo* and suggests short dialdehydes as putative end-product (Fig 8).

Methylglyoxal and glyoxal represent ubiquitous highly reactive compounds formed both enzymatically and non-enzymatically [105,106]. For instance, the majority of methylglyoxal is generated as a glycolytic by-product. Cellular detoxification systems effectively convert methylglyoxal/glyoxal into less-toxic metabolites and thus are capable to maintain tolerable steady-state levels. Intriguingly, methylglyoxal is detoxified into pyruvate, which can enter the tricarboxylic acid cycle. Accordingly, it might be possible that carotenoid oxidation allows recycling of carotenoid-derived carbon for primary metabolism upon truncation into C2 and C3 metabolites. This process might not be restricted to calli with an artificially high pathway activity through *PSY* overexpression. It is conceivable that this process represents a ubiquitous recycling mechanism for carotenoid-derived carbon as observed in WT calli as well as leaves and fruits. This would be similar to the vitamin E recycling process in which oxidized α-tocopherol is re-generated upon its oxidation to tocopherolquinone [107]. Further research is required to confirm whether carotenoids undergo carbon recycling.

### Conditions for carotenoid crystallization in plastids

In contrast to orange calli with strongly increased pathway activity (*At12* and *At22*), *PSY*-overexpressing lines with only about two-fold increased pathway activity (*At13* and *AtU16*) did not over-accumulate β-carotene and β-apocarotenal and carotene-dialdehyde levels were unchanged. We cannot fully rule out that increased pathway activity in weak expressors is compensated by increased turnover of xanthophylls. Xanthophyll turnover remains enigmatic since glycosylated xanthophyll-derived cleavage products which are known degradation routes in leaves, are absent in calli [13]. Thus, given the correlation between β-carotene over-accumulation and β-apocarotenals/apocarotene-dialdehyde formation, oxidative β-carotene destruction might be capable of keeping pace with higher β-carotene formation induced by slightly enhanced pathway activity. This maintains low steady-state levels similar to those in WT. Further increased pathway activity apparently outcompetes degradation and upon exceeding a critical concentration β-carotene over-accumulates and crystallizes in an autonomous process (Fig 9).

Upon biosynthetic arrest by NFZ, crystalline β-carotene decays with an average daily rate of 13 μg g^-1^. This disagrees with the high stability of carotenoid crystals concluded from their prevalence in fruits and vegetables. Differences in oxygen accessibility as well as concentrations of alternative antioxidants, as discussed above, might be capable in stabilizing carotenoid crystals. However, importantly, carotenoid crystals are observed in vital tissues with an active metabolism; thus high steady-state carotenoid levels might require an active carotenoid biosynthesis to compensate degradation.

In conclusion, the Arabidopsis callus system unravelled that biosynthesis and non-enzymatic degradation are critical pathway steps towards maintaining high steady-state β-carotene levels. Future investigations can consider the introduction of alternative antioxidants and/or the optimization of pathway enzymes in addition to PSY to further increase β-carotene synthesis above the rate of degradation. For instance, endosperm carotenoid content in Golden Rice lines differed dramatically only by using an array of PSY enzymes from different plant origins [61]. A systematic approach determining or even engineering PSY variants with highest performance *in planta* using the Arabidopsis callus system prior to their application could accelerate developing crops with further increased carotenoid contents. Moreover, combinatorial approaches of *PSY* overexpression with variants of the OR protein, MEP pathway enzymes or carotene hydroxylases would allow conclusions on promising gene editing applications in crops. Thus, the observed similarities of carotenoid properties in Arabidopsis calli with those in other carotenoid-rich crop tissues opens new doors towards improving provitamin A biofortification in crops.

## Materials and methods

### Plant materials

Arabidopsis AGI numbers and mutant alleles used are as follows: *NCED2* (At4g18350, SALK_090937, *nced2-3*); *NCED3* (At3g14440, N3KO-6620); *NCED5* (At1g30100, N5KO-4250); *NCED6* (At3g24220, WISC.DSLox471G6); *NCED9* (At1g78390, SALK_051969); *CCD1* (At3g63520, SAIL_390_C01, *ccd1-1*); *CCD4* (At4g19170, SALK_097984, *ccd4-1*); *CCD7* (At2g44990, *max3-11*); *CCD8* (At4g32810, *max4-6*); *CYP97C1* (At3g53130, SALK_129724C, *lut1*); *CYP97A3* (At1g31800, *lut5-1*); *PDS* (At4g14210, ZHJ070204); *CRTISO* (At1g06820, *ccr2-1*). For *35S*::*CrtI* lines, see [58]; for *35S*::*AtPSY* and *35S*::*ZmPSY1* lines, see [60].

### qPCR

RNA extraction, qPCR analysis and Arabidopsis PSY immunoblotting was performed as described in [44].

### Callus generation

Leaves from plants grown aseptically on MS agar were cut into pieces and incubated on CIM (4.33 g L^-1^ MS basal salts/KOH, pH 5.8, 3% [w/v] sucrose, 0.1% [v/v] Gamborg B5 vitamins, 0.5 mg L^-1^ 2,4-D, 2 mg L^-1^ indole-3-acetic acid, 0.5 mg L^-1^ 2-isopentenyladenine, 0.4% [w/v] phytagel) for four weeks. For seed-derived calli, ten milligrams of surface-sterilized seeds were plated onto petri dishes (145 mm diameter) containing CIM, germinated under long-day conditions (16 h light/8 h dark, 26°C) for five days and incubated for 14 days in darkness. For NFZ and trolox (6-hydroxy-2,5,7,8-tetramethylchroman-2-carboxylic acid) treatments, seeds were germinated on a filter paper on CIM medium, germinated as above and transferred to CIM medium supplemented with 1 μM NFZ or 500 μM trolox prior to etiolation.

### Carotenoid analysis

Callus carotenoids were extracted and analyzed as described in [108]. For saponification, extracts were mixed with 4 ml of ethanol and 120 μl KOH (1 g ml^-1^), heated for 10 min at 85°C and cooled. 6 ml 1% (w/v) NaCl solution was added and carotenoids were extracted as above.

### Apocarotenoid analysis

Analysis of retinal, β-apo-14’-carotenal, β-apo-12’-carotenal, β-apo-10’-carotenal, β-apo-8’-carotenal, β-apo-13-carotenone and β-ionylidene acetaldehyde was performed by LC-MS as described [38]. Extracts were spiked with an internal standard mix (75 pmol of each D3-β-apocarotenoid; Buchem, Netherlands) and with 50 μg α-tocopheryl-acetate. Apocarotenoids were subjected to atmospheric pressure chemical ionization (APCI) and analyzed in the positive mode. For quantification, the respective D3-labelled compounds served as internal standards. Standard curves were obtained with each unlabelled apocarotenoid (BASF, Germany) in a range of 0.5 − 15 pmol on-column containing a constant amount of 3 pmol of the respective D3-labelled compound. The TraceFinder 3.2 software (Thermo Fisher Scientific) was used for quantification based on the MS^1^ signal, the MS^2^ spectra serving as a qualifier. Peak areas of the photometric signals at 285 nm were integrated for α-tocopheryl-acetate and used to correct for unspecific losses during sample processing. Geronic acid was analyzed as described [38].

Upon β-apocarotenoid analysis, extracts were processed as follows for subsequent analysis of β-cyclocitral, β-ionone, 5,6-epoxy-β-ionone and apocarotene-dialdehydes. Samples were dissolved in 300 μl dichlormethane and derivatized by adding 15 μl of 200 μM 2,4-dinitrophenylhydrazine phosphoric acid solution (DNPH; Sigma-Aldrich) and incubating for 3 h at 37°C. After washing with 1 ml of water, the organic phase was recovered, dried under vacuum, redissolved in 50 μl dichlormethane:methanol (1:1, v/v) and 2 μl were subjected to LC-MS analysis. LC-MS parameters were identical as above except that APCI ionization was performed in negative mode. Derivatization efficiency was monitored by measuring underivatized D3-labelled apocarotenoids. D3-β-apo-14’-carotenal-DNPH was used as internal standard for normalization of apocarotene-dialdehyde signals. For analytical specificities of dinitrophenylhydrazone derivatives of β-ionone, β-cyclocitral, 5,6-epoxy-β-ionone and apocarotene-dialdehydes, see S2 Table. Methylglyoxal and glyoxal identity was confirmed by authentic standards (Sigma-Aldrich).

### *In vitro* phytoene synthase activity

Plastid were isolated from calli etiolated for two weeks according to [109]. Following lysis and centrifugation (21000*g for 10 min), the membranes were resuspended in lysis buffer and protein concentration was determined (Bio-Rad Protein Assay; Bio-Rad, Munich). For *in vitro* phytoene synthesis, 200 μg protein was incubated with 10 μM [^14^C]-isopentenyl diphosphate (IPP, 50 mCi mmol^-1^; ARC), 20 μM IPP (Isoprenoids), 40 μM dimethylallyl diphosphate, 4 mM ATP and 10 μg of recombinant, purified geranylgeranyl diphosphate synthase [110] for 30 min. Incubation conditions, product analysis and quantification was performed by thin layer chromatography and scintillation counting as described [17].

### Microscopy

Genetic crosses were made between a *AtPSY*-overexpressing line [44] and a line carrying a cyan fluorescent protein (CFP) labelled plastid-localized protein (pt-ck; CS16265) [65]. Callus protoplasts were prepared according to [111]. Fluorescence was imaged using a laser scanning microscope (LSM; Zeiss LSM 510 META). CFP fluorescence was excited with 458 nm, and emission was recorded at 475/525 nm. Carotenoid crystals were visualized using a 543 nm laser.

For TEM Arabidopsis roots from WT and line *At12* were generated [60], cut in approximately 1 mm long pieces, processed and analyzed using microtubule-stabilizing buffer and applying vacuum infiltration during fixation [112].

## Supporting information

S1 Fig Identification of xanthophyll esters in WT callus extracts. Arabidopsis seeds were germinated for 5 days on callus-inducing medium and incubated in darkness for 14 days. Carotenoids were extracted and an aliquot was saponified. HPLC contour plot (A) and a chromatogram at 450 nm (B) is shown between 50 and 77 min running time. C, Identical absorption spectra of one abundant xanthophyll ester (RT: 67 min) and antheraxanthin (RT: 18 min) suggests this xanthophyll as the major constituent of the esters.
(PDF)

S2 Fig Images of calli from Arabidopsis mutants. Seedlings were germinated on CIM under long day conditions for 5 days, then etiolated for 14 days. Three representative mutant calli are shown next to wild-type calli. Calli treated with norflurazon (NFZ) were transferred on CIM plates containing 1 μM NFZ prior to etiolation. A, Calli from carotenoid cleavage enzyme mutants; B, calli from carotenoid pathway enzyme mutants and from CrtI-expressing lines. For mutant abbreviations, see text.
(PDF)

S3 Fig HPLC chromatograms of Arabidopsis WT callus extracts. Arabidopsis WT seeds were germinated for 5 days on callus-inducing medium, transferred onto fresh medium (black line) or medium with 1 μM norflurazon (NFZ, red line) and developed further for 14 days in darkness. HPLC chromatograms at 450 nm (top) and 287 nm (bottom) are shown and most abundant carotenoids are indicated. IST, internal standard.
(PDF)

S4 Fig Images of Arabidopsis calli from different *AtPSY*-overexpressing lines. Seedlings were germinated on CIM under long day conditions for 5 days, then etiolated for 14 days. Calli treated with norflurazon (NFZ) were transferred on CIM plates containing 1 μM NFZ prior to etiolation. Three representative calli from transgenic lines are shown each.
(PDF)

S5 Fig Geronic acid amounts in WT and *ZmPSY1*-expressing calli. Calli from Arabidopsis WT and one *ZmPSY1*-expressing line were subjected to geronic acid (GA) extraction and analyzed by LC-MS. Geronic acid is liberated from non-enzymatically formed carotenoid-oxygen copolymers and considered as quantitative indicator for b-carotene oxidation products. Results are means ± SD from three biological replicates. Significant difference to the WT, *P<0.05.
(PDF)

S1 Table Detailed carotenoid contents in Arabidopsis calli for diagrams in Figs 2–4. Carotenoid amounts shown in Figs 2–4 are given in μg g DM^-1^ below; n.d., not detected; for other abbreviations, see figure legends.
(PDF)

S2 Table Analytical specificities of short-chain β-apocarotenals and apocarotene-dialdehydes. Short-chain β-apocarotenals (β-cyclocitral, β-ionone and 5,6-epoxy-β-ionone) and apocarotene-dialdehydes were analyzed by LC-MS as carbonyl dinitrophenylhydrazones after derivatization with 2,4-dinitrophenylhydrazine phosphoric acid. Chemical structures, formulas, retention times (RT) and extracted mass values for the corresponding carbonyl dinitrophenylhydrazones (DNPH) are given. Names correspond to underivatized compounds; C10, C08, C05, C03 and C02 depict apocarotene-dialdehydes with corresponding IUPAC names given below.
(PDF)
