# Supplementary material for: Establishment of an Arabidopsis callus system to study the interrelations of biosynthesis, degradation and accumulation of carotenoids
Source: PLoS One. 2018 Feb 2;13(2):e0192158. doi: 10.1371/journal.pone.0192158 (PMC5796706; doi:10.1371/journal.pone.0192158)
Supplement: S1 Fig — Arabidopsis seeds were germinated for 5 days on callus-inducing medium and incubated in darkness for 14 days. Carotenoids were extracted and an aliquot was saponified. HPLC contour plot (A) and a chromatogram at 450 nm (B) is shown between 50 and 77 min running time. C, Identical absorption spectra of one abundant xanthophyll ester (RT: 67 min) and antheraxanthin (RT: 18 min) suggests this xanthophyll as the major constituent of the esters. (PDF) [file pone.0192158.s001.pdf]

Supplemental Figure S1

A. Contour plot

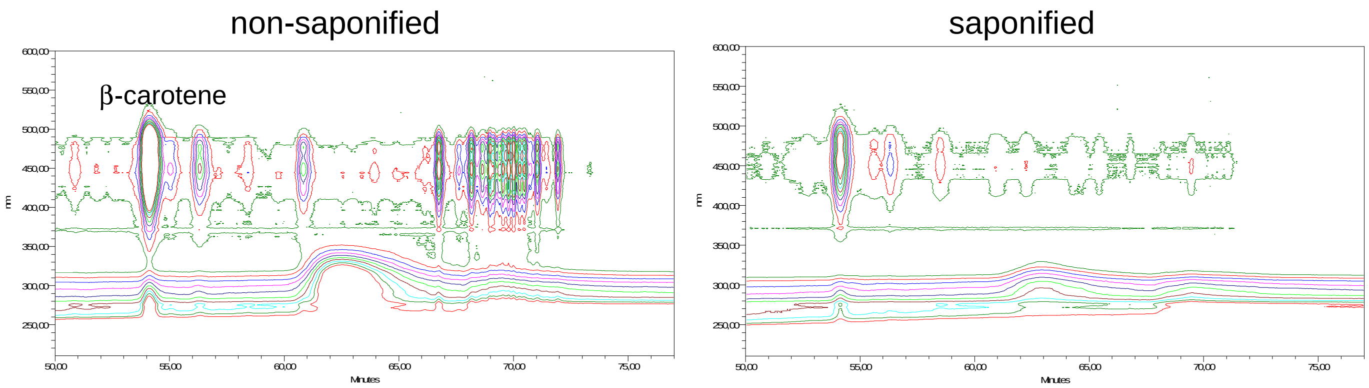

B. Chromatogram (450 nm)

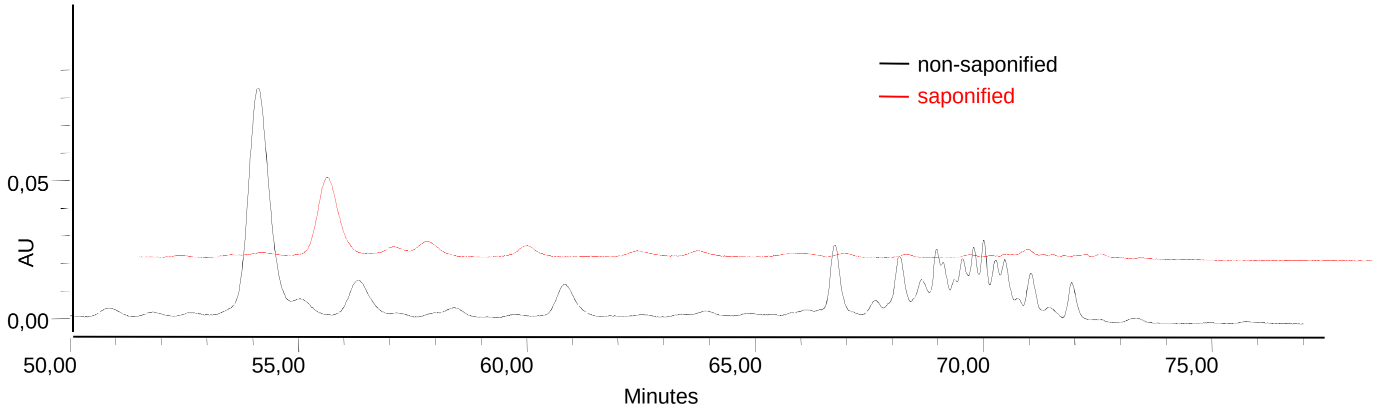

C. Absorption spectra

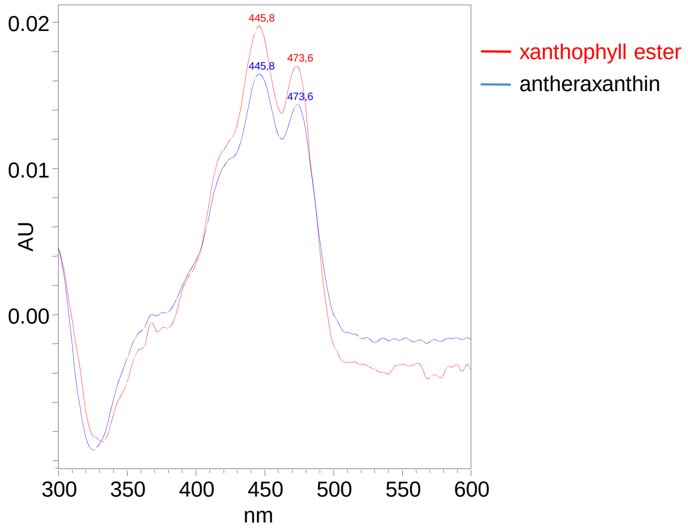

Supplemental Figure S1: Identification of xanthophyll esters in wild-type callus extracts

Arabidopsis seeds were germinated for 5 days on callus-inducing medium and incubated in darkness for 14 days. Carotenoids were extracted and an aliquot was saponified. HPLC contour plot (A) and a chromatogram at 450 nm (B) is shown between 50 and 77 min running time. C, Identical absorption spectra of one abundant xanthophyll ester (RT: 67 min) and antheraxanthin (RT: 18 min) suggests this xanthophyll as the major constituent of the esters.
