# Supplementary material for: Establishment of an Arabidopsis callus system to study the interrelations of biosynthesis, degradation and accumulation of carotenoids
Source: PLoS One. 2018 Feb 2;13(2):e0192158. doi: 10.1371/journal.pone.0192158 (PMC5796706; doi:10.1371/journal.pone.0192158)
Supplement: S2 Fig — Seedlings were germinated on CIM under long day conditions for 5 days, then etiolated for 14 days. Three representative mutant calli are shown next to wild-type calli. Calli treated with norflurazon (NFZ) were transferred on CIM plates containing 1 μM NFZ prior to etiolation. A, Calli from carotenoid cleavage enzyme mutants; B, calli from carotenoid pathway enzyme mutants and from CrtI-expressing lines. For mutant abbreviations, see text. (PDF) [file pone.0192158.s002.pdf]

Supplemental Figure S2

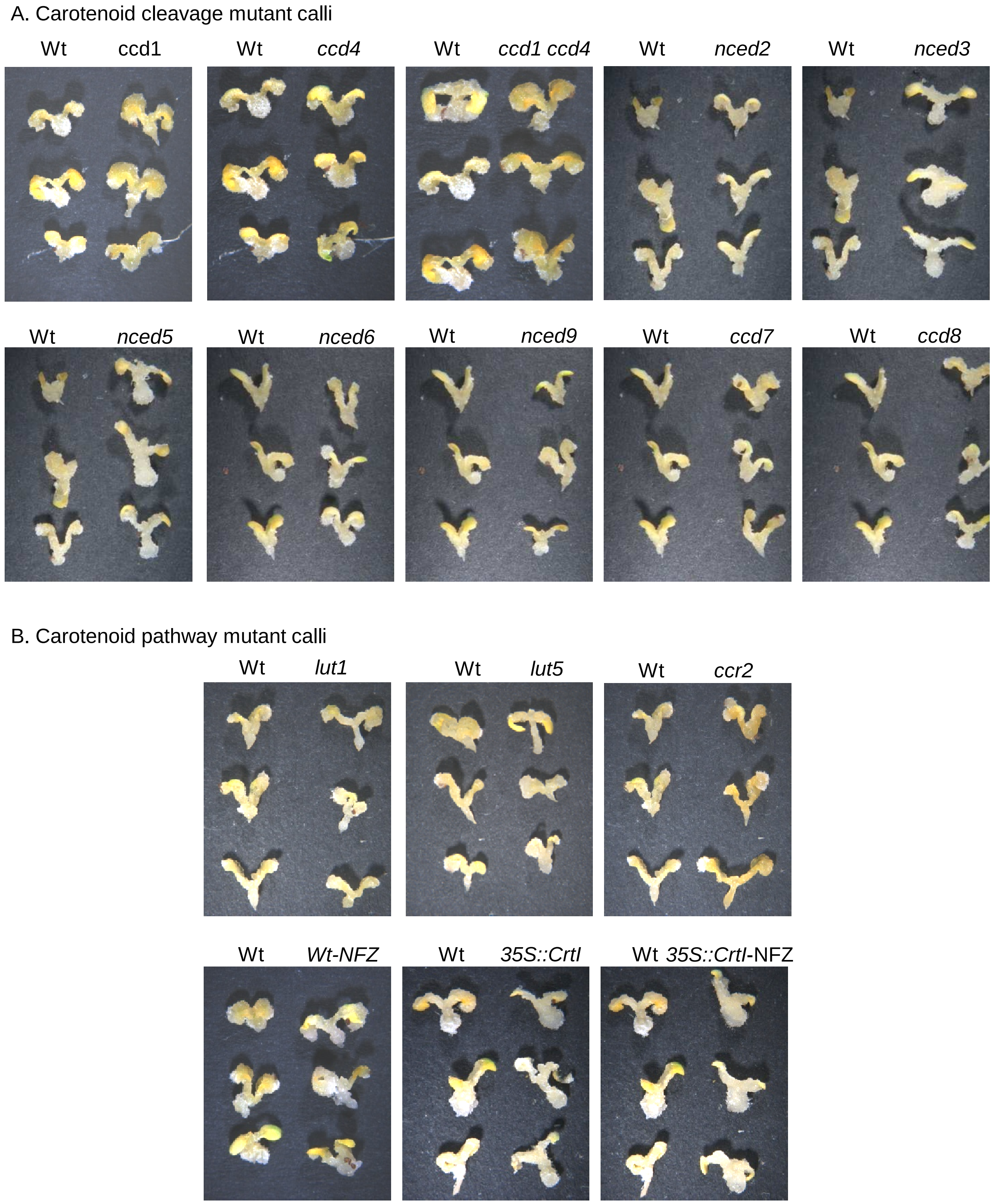

Supplemental Figure S2: Images of calli from Arabidopsis mutants.
Seedlings were germinated on CIM under long day conditions for 5 days, then etiolated for 14 days. Three representative mutant calli are shown next to wild-type calli. Calli treated with norflurazon (NFZ) were transferred on CIM plates containing 1 µM NFZ prior to etiolation. A, Calli from carotenoid cleavage enzyme mutants; B, calli from carotenoid pathway enzyme mutants and from CrtI-expressing lines. For mutant abbreviations, see text.
