# Supplementary material for: Establishment of an Arabidopsis callus system to study the interrelations of biosynthesis, degradation and accumulation of carotenoids
Source: PLoS One. 2018 Feb 2;13(2):e0192158. doi: 10.1371/journal.pone.0192158 (PMC5796706; doi:10.1371/journal.pone.0192158)
Supplement: S3 Fig — Arabidopsis WT seeds were germinated for 5 days on callus-inducing medium, transferred onto fresh medium (black line) or medium with 1 μM norflurazon (NFZ, red line) and developed further for 14 days in darkness. HPLC chromatograms at 450 nm (top) and 287 nm (bottom) are shown and most abundant carotenoids are indicated. IST, internal standard. (PDF) [file pone.0192158.s003.pdf]

Supplemental Figure S3

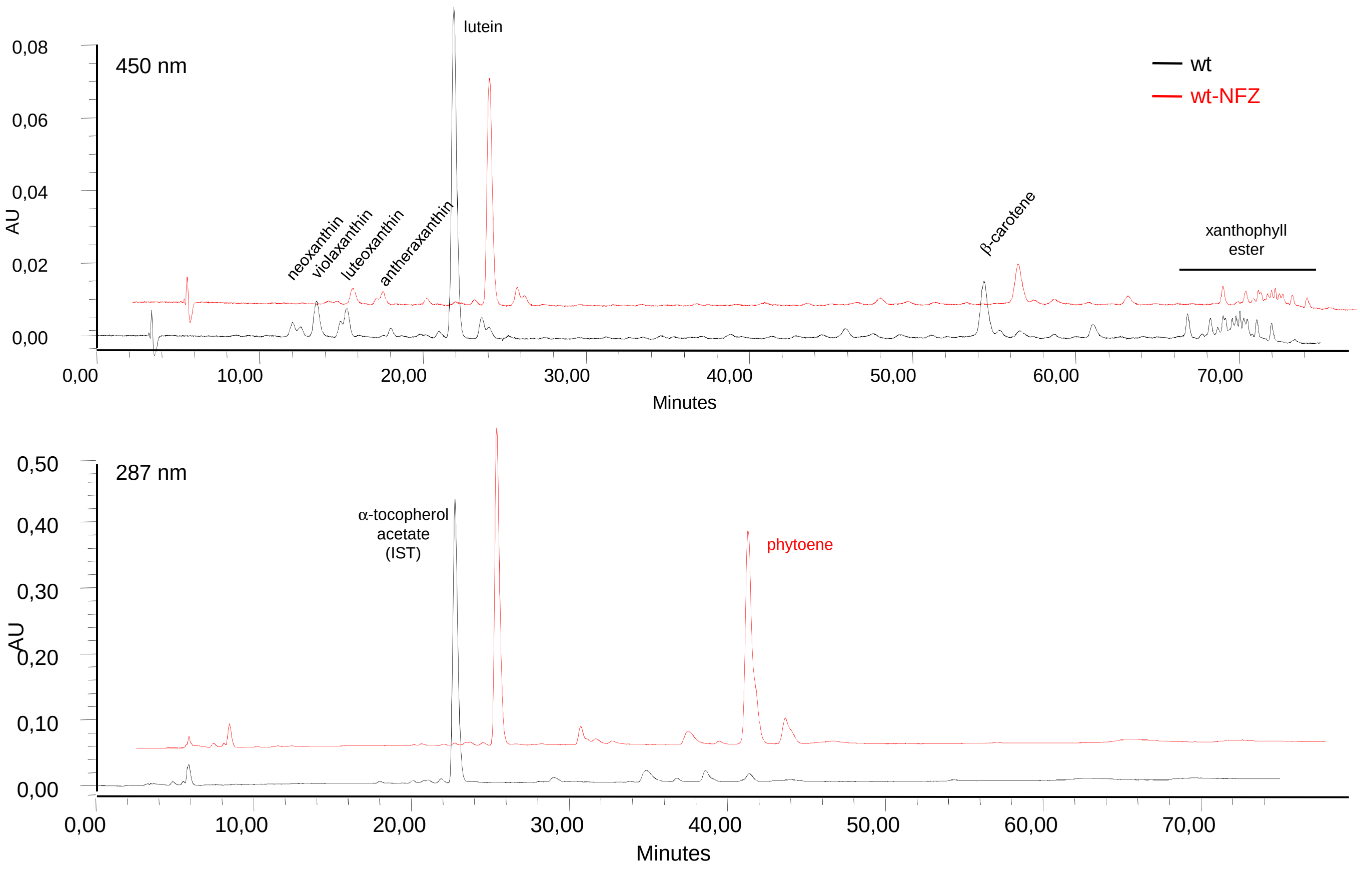

Supplemental Figure S3: HPLC chromatograms of Arabidopsis WT callus extracts
Arabidopsis WT seeds were germinated for 5 days on callus-inducing medium, transferred onto fresh medium (black line) or medium with 1 µM norflurazon (NFZ, red line) and developed further for 14 days in darkness. HPLC chromatograms at 450 nm (top) and 287 nm (bottom) are shown and most abundant carotenoids are indicated. IST, internal standard.
