# Supplementary material for: Establishment of an Arabidopsis callus system to study the interrelations of biosynthesis, degradation and accumulation of carotenoids
Source: PLoS One. 2018 Feb 2;13(2):e0192158. doi: 10.1371/journal.pone.0192158 (PMC5796706; doi:10.1371/journal.pone.0192158)
Supplement: S4 Fig — Seedlings were germinated on CIM under long day conditions for 5 days, then etiolated for 14 days. Calli treated with norflurazon (NFZ) were transferred on CIM plates containing 1 μM NFZ prior to etiolation. Three representative calli from transgenic lines are shown each. (PDF) [file pone.0192158.s004.pdf]

Supplemental Figure S4

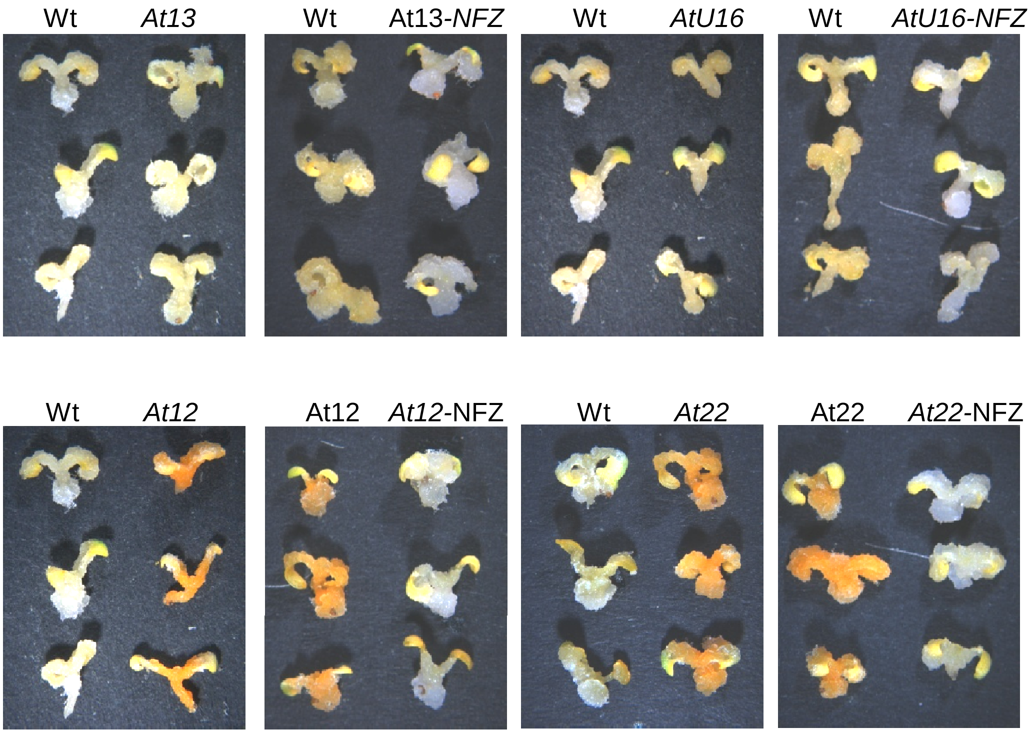

Supplemental Figure S4: Images of Arabidopsis calli from different *AtPSY*-overexpressing lines
Seedlings were germinated on CIM under long day conditions for 5 days, then etiolated for 14 days. Calli treated with norflurazon (NFZ) were transferred on CIM plates containing 1 µM NFZ prior to etiolation. Three representative calli from transgenic lines are shown each.
