# Supplementary material for: Establishment of an Arabidopsis callus system to study the interrelations of biosynthesis, degradation and accumulation of carotenoids
Source: PLoS One. 2018 Feb 2;13(2):e0192158. doi: 10.1371/journal.pone.0192158 (PMC5796706; doi:10.1371/journal.pone.0192158)
Supplement: S5 Fig — Calli from Arabidopsis WT and one ZmPSY1-expressing line were subjected to geronic acid (GA) extraction and analyzed by LC-MS. Geronic acid is liberated from non-enzymatically formed carotenoid-oxygen copolymers and considered as quantitative indicator for b-carotene oxidation products. Results are means ± SD from three biological replicates. Significant difference to the WT, *P<0.05. (PDF) [file pone.0192158.s005.pdf]

Supplemental Figure S5

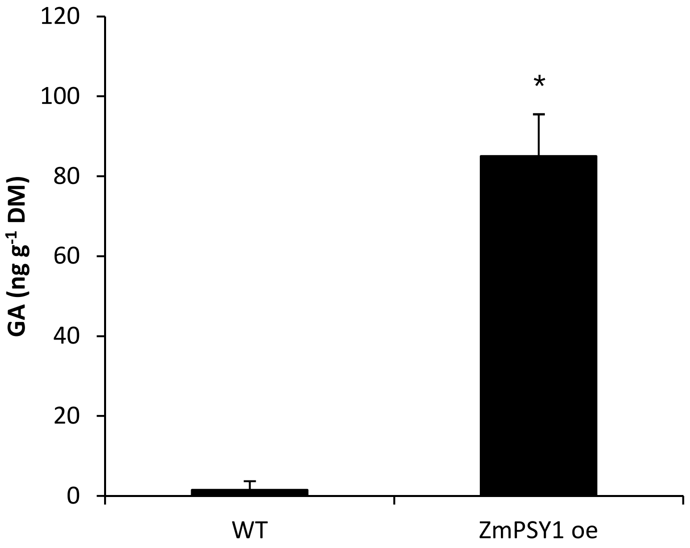

Supplemental Figure S5: Geronic acid amounts in WT and *ZmPSY1*-expressing calli
Calli from Arabidopsis WT and one *ZmPSY1*-expressing line were subjected to geronic acid (GA) extraction and analyzed by LC-MS. Geronic acid is liberated from non-enzymatically formed carotenoid-oxygen copolymers and considered as quantitative indicator for β-carotene oxidation products. Results are means ± SD from three biological replicates. Significant difference to the WT, *P<0.05.
