# Supplementary material for: Establishment of an Arabidopsis callus system to study the interrelations of biosynthesis, degradation and accumulation of carotenoids
Source: PLoS One. 2018 Feb 2;13(2):e0192158. doi: 10.1371/journal.pone.0192158 (PMC5796706; doi:10.1371/journal.pone.0192158)
Supplement: S1 Table — Carotenoid amounts shown in Figs 2–4 are given in μg g DM-1 below; n.d., not detected; for other abbreviations, see figure legends. (PDF) [file pone.0192158.s006.pdf]

Supplemental Table S1: Carotenoid levels in callus

Carotenoid amounts shown in figures 2-4 are given in µg g DM^{-1} below; n.d., not detected; for other abbreviations, see figure legends.

Carotenoid amounts, Fig. 2A

**sample**	**total carotenoids**	**viola+neox**	**other xanthos**	**lutein**	**α-/β-carotene**
**lvs**	2278.4 ± 88.7	773.0 ± 38.0	127.3 ± 5.5	1056.8 ± 71.9	321.3 ± 21.0
**lvs (callus 28d)**	40.8 ± 2.6	6.9 ± 0.8	15.0 ± 2.0	14.7 ± 2.7	4.2 ± 1.4
**sdl**	1416.3 ± 69.6	382.0 ± 40.0	138.9 ± 16.7	689.0 ± 55.4	206.4 ± 13.4
**Wt-7d**	543.4 ± 42.8	107.5 ± 7.0	91.8 ± 13.4	294.6 ± 17.2	49.5 ± 5.1
**Wt-14d**	181.3 ± 13.8	21.3 ± 3.2	72.0 ± 6.3	67.6 ± 6.3	24.0 ± 3.0

Carotenoid amounts, Fig. 2B

sample	total carotenoids	other xanthos	lutein	α-/β-carotene
**Wt**	198.3 ± 8.8	91.8 ± 12.1	79.9 ± 5.0	26.7 ± 8.1
ccd1	363.6 ± 7.4*	161.9 ± 8.3*	156.5 ± 7.0*	45.1 ± 5.1*
ccd4	424.3 ± 34.7*	217.9 ± 14.2*	120.1 ± 8.3*	86.3 ± 12.2*
ccd1-4	391.1 ± 19.4*	205.3 ± 6.8*	131.8 ± 3.8*	53.9 ± 10.3*
nced2	169.2 ± 19.4	77.5 ± 3.3	65.2 ± 11.3	24.9 ± 6.5
nced3	216.0 ± 12.2	97.4 ± 3.5	92.0 ± 13.6	24.3 ± 3.4
nced5	191.8 ± 10.7	94.3 ± 3.5	71.4 ± 7.1	24.2 ± 3.9
nced6	199.8 ± 15.8	85.7 ± 15.0	72.6 ± 8.3	39.4 ± 9.4
nced9	214.0 ± 46.1	97.3 ± 17.2	81.0 ± 22.1	33.8 ± 10.4
ccd7	175.2 ± 10.7	76.5 ± 9.7	62.8 ± 7.1	34.1 ± 6.1
ccd8	225.4 ± 6.0	104.6 ± 7.7	90.0 ± 10.6	28.8 ± 3.8
**Wt+Trol**	278.3 ± 16.2*	125.9 ± 5.8*	113.8 ± 9.5*	38.0 ± 6.7

Results are mean ± SD from at least three biological replicates. Significant difference to the WT, *P<0.05.

Carotenoid amounts, Fig. 3A

sample	total carotenoids	other xanthos	lutein	α-/β-carotene	phytoene	phyto-fluene	ζ-carotene	pro-lycopene	neuro-sporene	zeino-xanthin
**Wt**	201.4 ± 6.8	93.2 ± 7.2	75.3 ± 3.1	31.4 ± 6.8	n.d.	n.d.	n.d.	n.d.	n.d.	n.d.
lut1	87.9 ± 18.3*	70.9 ± 16.7	0.0 ± 0.0*	3.6 ± 0.2*	n.d.	n.d.	n.d.	n.d.	n.d.	13.5 ±1.3*
lut5	183.9 ± 23.7	83.0 ± 1.6	60.0 ± 15.8	41.0 ± 9.0	n.d.	n.d.	n.d.	n.d.	n.d.	n.d.
ccr2	400.9 ± 24.6*	102.7 ± 19.4	19.9 ± 2.4*	31.0 ± 2.5	61.3 ± 3.2*	47.0 ± 4.3*	75.9 ± 5.1*	38.0 ± 5.7*	20.4 ± 3.5*	n.d.

Results are mean ± SD from at least three biological replicates. Significant difference to the WT, *P<0.05.

Carotenoid amounts, Fig. 3B

sample	total carotenoids	other xanthos	lutein	α-/β-carotene	phytoene	phytofluene
**Wt-7d**	543.4 ± 42.8	199.3 ± 20.5	294.6 ± 17.2	49.5 ± 5.1	n.d.	n.d.
**Wt-7d+NFZ**	733.8 ± 51.2	215.3 ± 13.4	349.6 ± 30.6	65.5 ± 5.0	103.4 ± 22.4*	n.d.
**Wt-14d**	211.75 ± 61.0	108.2 ± 10.2	80.4 ± 8.6	29.7 ± 4.3	n.d.	n.d.
**Wt-14d+NFZ**	698.7 ± 99.2*	94.4 ± 17.4	95.3 ± 12.5	40.5 ± 5.6	465.8 ± 99.0*	30.9 ± 12.9*
***ccr2*+NFZ**	578.8 ± 45.7	71.1 ± 12.2	34.1 ± 0.7*	46.0 ± 12.4	392.8 ± 3.5	28.9 ± 2.1

Results are mean ± SD from at least three biological replicates. Significance was tested for wt samples of same age (7/14 d) and for NFZ-treated *ccr2* against Wt-14d+NFZ (*P<0.05).

Carotenoid amounts, Fig. 3C

sample	total carotenoids	other xanthos	lutein	α-/β-carotene	phytoene	phytofluene
**Wt-14d+NFZ**	718.2 ± 9.2	69.3 ± 11.8	44.5 ± 6.7	19.0 ± 4.5	536.9 ± 10.5	46.5 ± 3.4
***ccd1*+NFZ**	795.1 ± 22.7	101.8 ± 8.7	103.2 ± 14.1	21.6 ± 1.1	502.7 ± 28.8	65.8 ± 1.8*
***ccd4*+NFZ**	680.3 ± 131.3	89.2 ± 27.5	75.8 ± 7.5	30.0 ± 2.7	444.4 ± 94.1	40.9 ± 14.6
***ccd1-4*+NFZ**	779.6 ± 4.4*	77.1 ± 6.8	79.8 ± 6.6	20.3 ± 0.9	548.9 ± 3.7	53.6 ± 1.8
pds	585.9 ± 63.4*	n.d.	n.d.	n.d.	585.9 ± 63.4	n.d.
**Wt-14d**	201.4 ± 6.8	93.2 ± 7.2	75.3 ± 3.1	31.4 ± 6.8	n.d.	n.d.
35S:CrtI	210.8 ± 10.9	114.9 ± 7.2	67.1 ± 2.9	28.7 ± 2.2	n.d.	n.d.
***35S:CrtI*+NFZ**	223.3 ± 10.8	118.3 ± 4.9	75.2 ± 5.0	29.8 ± 1.7	n.d.	n.d.

Results are mean ± SD from at least three biological replicates. Significance was tested for *pds* and NFZ-treated *ccd* mutants against NFZ-treated Wt control (*P<0.05). Difference of *35S:CrtI* and *35S:CrtI*+NFZ to Wt-14d was non-significant (P<0.05).

Carotenoid amounts, Fig. 4A

sample	total carotenoids	other xanthos	lutein	α-/β-carotene	phytoene	phytofluene
**Wt-14d**	211.7 ± 17.6	108.2 ± 10.2	80.4 ± 8.6	29.7 ± 4.3	n.d.	n.d.
**Wt-14d+NFZ**	715.7 ± 43.1	89.9 ± 10.2	90.4 ± 16.0	32.5 ± 7.6	485.9 ± 43.6	33.2 ± 8.3
At13	224.2 ± 14.8	103.5 ± 6.1	93.6 ± 8.4	27.2 ± 5.3	n.d.	n.d.
***At13*+NFZ**	1461.8 ± 40.5*	88.9 ± 20.3	79.0 ± 7.8	31.8 ± 8.3	1236.8 ± 25.9*	25.3 ± 10.3
AtU16	315.9 ± 7.5*	152.8 ± 2.1	133.5 ± 3.9*	29.6 ± 2.6	n.d.	n.d.
***AtU16*+NFZ**	1577.9 ± 22.3*	66.2 ± 8.3	46.4 ± 11.1	9.5 ± 4.1	1420.5 ± 27.9*	35.3 ± 20.4
At12	1551.7 ± 70.5*	201.9 ± 27.2*	160.6 ± 11.5*	776.9 ± 86.5*	278.2 ± 45.3*	137.8 ± 9.0*
***At12*+NFZ**	2252.8 ± 270.9*	116.8 ± 30.9	141.0 ± 22.9	65.0 ± 16.5	1882.5 ± 208.7*	47.5 ± 24.4
At22	1220.9 ± 32.0*	174.6 ± 29.6*	177.5 ± 24.5*	554.6 ± 77.7*	240.9 ± 61.7*	73.3 ± 20.1*
***At22*+NFZ**	2247.3 ± 50.0*	97.1 ± 9.2	99.6 ± 23.6	102.5 ± 86.6	1933.5 ± 89.3*	14.6 ± 14.6

Results are mean ± SD from at least three biological replicates. Significance was tested against non-treated and NFZ-treated Wt controls, respectively (*P<0.05).
