# Supplementary material for: Establishment of an Arabidopsis callus system to study the interrelations of biosynthesis, degradation and accumulation of carotenoids
Source: PLoS One. 2018 Feb 2;13(2):e0192158. doi: 10.1371/journal.pone.0192158 (PMC5796706; doi:10.1371/journal.pone.0192158)
Supplement: S2 Table — Short-chain β-apocarotenals (β-cyclocitral, β-ionone and 5,6-epoxy-β-ionone) and apocarotene-dialdehydes were analyzed by LC-MS as carbonyl dinitrophenylhydrazones after derivatization with 2,4-dinitrophenylhydrazine phosphoric acid. Chemical structures, formulas, retention times (RT) and extracted mass values for the corresponding carbonyl dinitrophenylhydrazones (DNPH) are given. Names correspond to underivatized compounds; C10, C08, C05, C03 and C02 depict apocarotene-dialdehydes with corresponding IUPAC names given below. (PDF) [file pone.0192158.s007.pdf]

Supplemental Table S2: Analytical specificities of short-chain β-apocarotenals and apocarotene-dialdehydes

Short-chain β-apocarotenals (β-cyclocitral, β-ionone and 5,6-epoxy-β-ionone) and apocarotene-dialdehydes were analyzed by LC-MS as carbonyl dinitrophenylhydrazones after derivatization with 2,4-dinitrophenylhydrazine phosphoric acid. Chemical structures, formulas, retention times (RT) and extracted mass values for the corresponding carbonyl dinitrophenylhydrazones (DNPH) are given. Names correspond to underivatized compounds; C10, C08, C05, C03 and C02 depict apocarotene-dialdehydes with corresponding IUPAC names given below.

**Name**	**Structure (DNPH)**	**Formula (DNPH)**	**RT (DNPH) [min]**	**Extracted mass DNPH [M – H]$^-$**
**β-Cyclocitral**	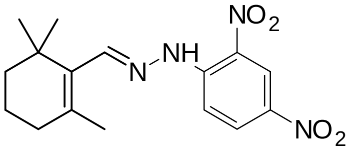	$C_{16}H_{20}N_4O_4$	5.1	331.1412
**β-Ionone**	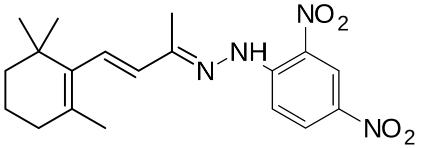	$C_{19}H_{24}N_4O_4$	6.4	371.1725
**5,6-Epoxy-β-ionone**	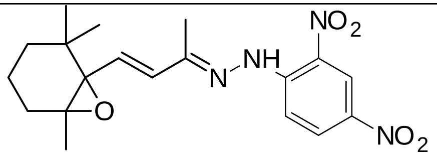	$C_{19}H_{24}N_4O_5$	4.8	387.1674
**C10** (2E,4E,6E)-2,6-dimethylocta -2,4,6-trienedial	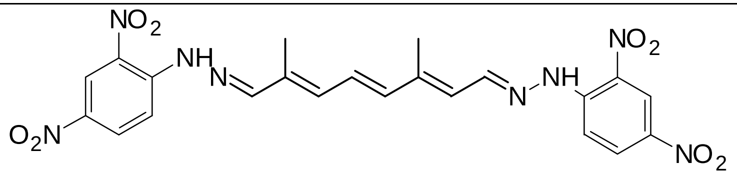	$C_{22}H_{20}N_8O_8$	5.1	523.1331

**C08**<br>(2E,4E)-2-methyl-6-oxohepta<br>-2,4-dienal	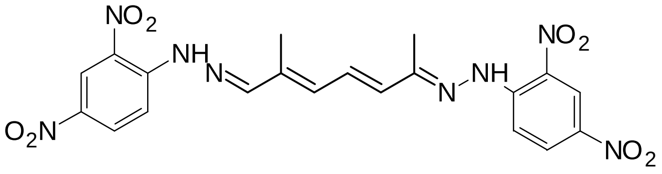	$C_{20}H_{18}N_8O_8$	4.5	497.1175
**C05**<br>(2E)-2-methylbut<br>-2-enedial	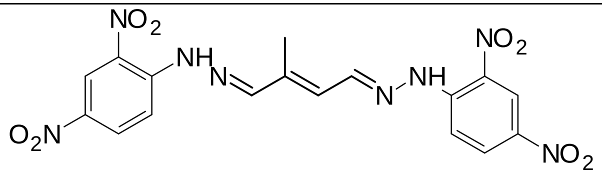	$C_{17}H_{14}N_8O_8$	3.1	457.0862
**C03** (methyl glyoxal)<br>2-oxopropanal	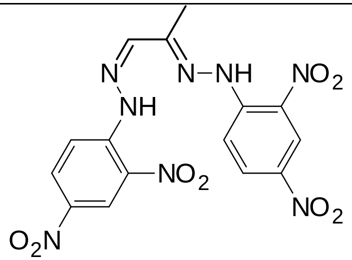	$C_{15}H_{12}N_8O_8$	2.5	431.0705
**C02** (glyoxal)<br>ethanedial	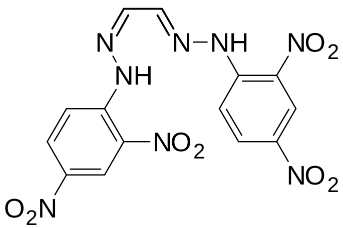	$C_{14}H_{10}N_8O_8$	2.0	417.0549
